# Isolation and characterisation of transport-defective substrate-binding mutants of the tetracycline antiporter TetA(B)

**DOI:** 10.1016/j.bbamem.2015.06.026

**Published:** 2015-10

**Authors:** David J. Wright, Christopher G. Tate

**Affiliations:** MRC Laboratory of Molecular Biology, Cambridge Biomedical Campus, Francis Crick Avenue, Cambridge CB2 0QH, UK

**Keywords:** Membrane protein, Transporter, Isothermal titration calorimetry, Conformational thermostabilisation

## Abstract

The tetracycline antiporter TetA(B) is a member of the Major Facilitator Superfamily which confers tetracycline resistance to cells by coupling the efflux of tetracycline to the influx of protons down their chemical potential gradient. Although it is a medically important transporter, its structure has yet to be determined. One possibility for why this has proven difficult is that the transporter may be conformationally heterogeneous in the purified state. To overcome this, we developed two strategies to rapidly identify TetA(B) mutants that were transport-defective and that could still bind tetracycline. Up to 9 amino acid residues could be deleted from the loop between transmembrane α-helices 6 and 7 with only a slight decrease in affinity of tetracycline binding as measured by isothermal titration calorimetry, although the mutant was transport-defective. Scanning mutagenesis where all the residues between 2 and 389 were mutated to either valine, alanine or glycine (VAG scan) identified 15 mutants that were significantly impaired in tetracycline transport. Of these mutants, 12 showed no evidence of tetracycline binding by isothermal titration calorimetry performed on the purified transporters. In contrast, the mutants G44V and G346V bound tetracycline 4–5 fold more weakly than TetA(B), with K_d_s of 28 μM and 36 μM, respectively, whereas the mutant R70G bound tetracycline 3-fold more strongly (K_d_ 2.1 μM). Systematic mutagenesis is thus an effective strategy for isolating transporter mutants that may be conformationally constrained and which represent attractive targets for crystallisation and structure determination.

## Highlights

• A rapid method was developed for the identification of transport-defective mutants of TetA(B).
• ITC was used to determine the affinity of tetracycline binding to the mutants.
• Fifteen transport-defective point mutations were identified.
• Three mutants bound tetracycline whereas the remainder did not
• The transport-defective mutants may facilitate crystallisation of TetA(B).

## Graphical abstract

**Figure:**
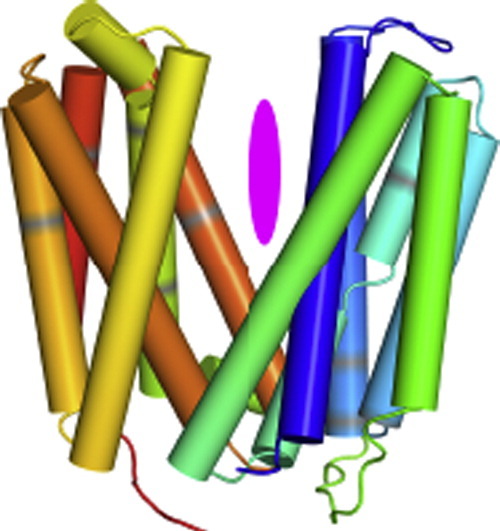

## 1 Introduction

Bacterial resistance to tetracycline arises from the expression of any one of at least 23 known tetracycline transporters, which couple export of tetracycline to the influx of protons down their electrochemical gradient [1]. Of these tetracycline antiporters, TetA(B), a member of the major facilitator superfamily (MFS), has the widest host range in Gram-negative bacteria. [2]. MFS transporters are comprised of twelve transmembrane α-helices arranged in two bundles of six helices (helices 1–6 and 7–12), which are thought to have arisen from a gene duplication that, in turn, was the result of a duplication of a three-helix motif [3]. Crystal structures of 17 members of the MFS are available in a number of conformations [4–29]. Generally, the approach taken has been to express hundreds of transporter homologs and to work on those that are most highly expressed and most stable in detergent, rather than the most therapeutically relevant. So, although the MFS family has been extensively studied, there are no available crystal structures for close homologs of TetA(B) and hence it is desirable that this transporter be crystallised and examined in molecular detail.

TetA(B) was the first drug resistance transporter discovered [30] and was shown to confer the exchange of a tetracycline-Mg^2 +^ adduct and a proton [31,32]. Each amino acid residue of TetA(B) has been systematically mutated to cysteine and the mutants were used to estimate the solvent exposure of side chains by measuring the ability of N-ethylmaleimide (NEM) to label the mutated transporter [33–39]. These data also identified mutations at 17 positions that resulted in a significant reduction in tetracycline resistance. Currently the only structural data available are from 2-dimensional crystals of TetA(B), but the data are at low resolution (~ 17 Å) [40]. 3-Dimensional crystals have not yet been reported, suggesting that the conformational dynamics and/or the stability of the detergent-solubilised transporter may be preventing the formation of well-diffracting crystals, and hence in this work an alternative strategy was tested in efforts to improve the probability of crystal formation.

One possibility to increase the likelihood of crystallising TetA(B) is to introduce mutations into the transporter that decrease conformational dynamics and also possibly improve thermostability. This strategy, termed conformational thermostabilisation [41], has been very successful for membrane protein crystallography, in particular for G protein-coupled receptors (GPCRs) [42,43]. The identification of mutations that result in a protein more resistant to thermal denaturation has been possible using a radioligand-binding assay, coupled to systematic mutant generation [42]. These mutations increased the stability of the protein in all detergents tested, thus allowing crystallisation trials to be performed in even short chain denaturing detergents such as nonylglucoside and octylthioglucoside [41]. Thermostabilising mutations in GPCRs selected using a radiolabelled antagonist resulted in a thermostabilised receptor preferentially found in an antagonist binding conformation, as shown by the affinity for antagonists that was unchanged, whereas the affinity for agonists was orders of magnitude weaker than for the wild type receptor. Therefore, selection using a radioligand-binding assay resulted in a membrane protein with increased thermostability and a conformational bias [42].

Conformational thermostabilisation has also been successful for the rat serotonin transporter (SERT) [44]. The thermostabilised mutant was over 17 °C more stable than the wild type transporter and it was locked in an outward-facing conformation that bound the cocaine-analogue RTI55 with high affinity and also the substrate serotonin with similar affinity to the wild type transporter. However, although the mutated transporter could bind inhibitors and substrate, it could not transport ^3^H-serotonin, suggesting that the transporter was indeed heavily biased towards a single conformation. Interestingly, the majority of the thermostabilising mutations were predicted to be at the interfaces between transmembrane α-helices and, in particular, within or adjacent to regions where the α-helices were kinked or were not helical.

One of the problems with conformational thermostabilisation is that the radioligand binding assays require high affinity natural substrates or inhibitors (in the nM range). However, the substrates of many secondary transporters, such as TetA(B), are of much lower affinity (μM–mM) and so the current methodology as applied to GPCRs will be difficult to implement with many transporters. There is therefore a need for a technique analogous to conformational thermostabilisation that can be employed for all secondary transporters. The approach used here was based on the observation that the thermostabilised serotonin transporter was incapable of functioning as a transporter, despite being able to bind substrate [44]. Thus selecting for mutants that prevented substrate transport but which still allowed substrate binding with an affinity similar to the wild type transporter would result in a transporter with properties more suitable for structural studies i.e. biased towards a single conformation.

Two methodologies were employed in this work to identify mutations that biased the conformation of TetA(B). Firstly, deletions in the non-conserved loop between transmembrane helices 6 and 7 were made to prevent the conformational change between outward-open and inward-open states. The second approach involved the selection of conformationally restrained mutants of TetA(B) using phenotypic selection, in combination with measurement of their expression levels. Loop deletion mutants and systematic point mutants were then analysed by isothermal titration calorimetry (ITC) to identify those mutants that retained substrate binding capabilities, despite being transport-defective, and therefore were likely to be conformationally restrained.

## 2 Materials and methods

### 2.1 Constructs

TetA(B) was cloned as a C-terminal enhanced GFP (eGFP) fusion in pBluescript SKII (+) in the orientation so that it was expressed from the *lac*UV5 promoter. PCR was performed with the templates transposon Tn10 and a gene fusion provided by Dr. J Andréll for TetA(B) and eGFP respectively in 0.2 ml thin-walled 8-tube strips (Thermoscientific), using KOD hot start polymerase (Novagen) and the manufacturer's protocol. Primers were designed to ligate eGFP between *Eco*RI and *Hind*III sites with T4 DNA ligase, before ligating TetA(B) between the *Sac*I and *Eco*RI sites, resulting in a transporter with a C-terminal eGFP fusion. This resulted in TetA(B)-eGFP, which was used for deletion mutagenesis.

The TetA(B) ITC constructs (TetA(B)-His10 or TetA(B)∆394–401-Thrombin-His10) were also inserted between the restriction sites *Nde*I and *Hind*III, but in plasmid pET21b under the control of the T7 promoter. The construct TetA(B)-Δ394–401-TEV-His10-eGFP was used for all the scanning mutagenesis experiments and was expressed from the *lac*UV5 promoter in pBluescript; the construct contained an additional C-terminal truncation of 8 residues with a tobacco etch virus (TEV) protease cleavage site and decahistidine tag (His10) between TetA(B) and eGFP, which were introduced by PCR using mismatched primers. Ligations were transformed into competent *Escherichia coli* XL1 cells, colonies grown for 18 h shaking at 250 rpm at 37 °C were picked, miniprepped and sequenced. Minipreps and maxipreps were performed using QIAGEN kits and the standard protocols provided. All restriction endonucleases and ligase were purchased from New England Biolabs. Plasmids were sequenced by Beckman Coulter Genomics using a synthesised primer complimentary to an internal site in eGFP (GGCCGTTTACGTCGCCGTCC) and standard primers complementary to the T7 promoter and M13 reverse. Sequencing typically achieved double coverage of the transporter and single coverage of the C-terminal eGFP.

### 2.2 Site directed mutagenesis of TetA(B)

Mutagenesis of TetA(B) was performed to identify mutations that were likely to increase crystallisation probability. OptimusPrimer 2.0 (propriety primer design software, Heptares Therapeutics) was used to design degenerate mutagenic primers for the introduction of mutations of 378 of the 401 residues (amino acids 2–379) of TetA(B). Each amino acid was individually mutated to valine, alanine or glycine (VAG) using the codon GBC, where B represents either C, G or T, resulting in GCC (Ala), GGC (Gly) or GTC (Val) introduced at a 1:1:1 ratio.

These VAG primers (produced in 96-well format by Integrated DNA Technologies) were used to introduce site-directed mutations into the template TetA(B)-Δ394–401-TEV-His10-eGFP in pBluescript SKII(+) by PCR using KOD hot start polymerase in 96-well thermowell (Costar) plates. Reaction mixtures included 1 x KOD hot start polymerase buffer, 0.2 mM of each dNTP, 1.5 mM MgSO_4_, 9% (v/v) DMSO, 1 U KOD hot start polymerase, template plasmid DNA at approximately 5 ng/μl and 0.2–0.4 μM of forward and reverse primers in a final 50 μl reaction volume. The PCR reaction consisted of an initial 5 min of melting at 95 °C, followed by 30–40 rounds of: melting at 95 °C for 30 s, annealing at 50–60 °C for 30 s and extension at 70 °C for 10 min. This was followed by 20–30 min at 70 °C to ensure all extension reactions were completed. The methylated template DNA was then digested by addition of 40 Units of *Dpn*I (New England Biolabs) by incubation for at least 5 h at 37 °C. 10 μl of each product was added to freshly thawed 200 μl *E. coli* JM109 cells in 96-well deep well blocks (VWR International Ltd), incubated on ice for 30 min before being heat shocked in a water bath for 90 s at 42 °C and incubated on ice for 5 min. 500 μl of SOB media, pre-warmed at 37 °C, was then added and the cells were incubated at 37 °C shaking at 220 rpm for 1 h. The cells were harvested by centrifugation, approximately 500 μl of the supernatant was removed and the cell pellet was resuspended in the remaining supernatant of approximately 200 μl. This was then plated on 2 × TY agar plates in either individual 90 mm or on large 7 × 7 divided formats containing 100 μg/ml ampicillin. Plates were then dried for 30–60 min and incubated for 18 h inverted at 37 °C.

For the introduction of truncations and deletions into TetA(B)-eGFP in pBluescript, TetA(B)His10 and TetA(B)-Δ394–401-Thrombin-His12 an identical PCR reaction to that above was used. The primers were designed manually with similar T_m_ values to those used in point mutagenesis; however nucleotides coding for residues to be truncated were omitted from forward and reverse primers. This was followed by *Dpn*I digestion and transformation into XL1 cells using the same protocol as listed above on individual 90 mm 2 × TY agar plates containing 100 μg/ml ampicillin. Multiple colonies were picked into 5 ml 100 μg/ml ampicillin in 2 × TY, grown for 18 h at 37 °C shaking at 220–250 rpm, miniprepped and sequenced.

### 2.3 Antibiotic resistance phenotypic assay of TetA(B) deletion mutants in liquid media

The phenotype of each truncation mutant in the TetA(B)-eGFP construct was analysed by growing cultures in liquid media to stationary phase and measuring the final attained OD_600_. Colonies of transformants in *E. coli* strain JM109 were picked into 5 ml 2 × TY media (16 g/l tryptone, 10 g/l yeast extract, 86 mM NaCl, pH 7.4) containing 100 μg/ml ampicillin and grown for 18 h at 37 °C shaking at 220 rpm. 5 μl of the overnight cultures was added to 1 ml of fresh 2 × TY media containing 100 μg/ml ampicillin and either 0 μM, 2 μM, 5 μM, 10 μM, 20 μM, 30 μM or 50 μM tetracycline (in triplicate) in 96-deep well plates and grown at 220 rpm at 37 °C for 18 h. 200 μl samples were transferred to a 96-well plate Nunclon flat-bottomed black plate (Sigma Aldrich) and the cell density (OD_600_) was measured using the Tecan Safire II and compared to JM109 cells transformed with wild type TetA(B)-eGFP in pBluescript SKII(+) as a positive control and pBluescript containing no insert as a negative control. Expression of TetA(B) relied on leaky expression from the *lac*UV5 promoter in the absence of IPTG.

### 2.4 High-throughput antibiotic resistance phenotypic assay of TetA(B) site-directed point mutants

From the bacterial colonies produced from VAG scanning mutagenesis, 8 colonies were picked for each amino acid position in order to, on average, pick at least one of each of alanine, glycine and valine-encoding codons. These colonies were picked into 1 ml 2 × TY media (16 g/l tryptone, 10 g/l yeast extract, 86 mM NaCl, pH 7.4) containing 100 μg/ml ampicillin in 96-well deep well blocks and grown for 18 h at 37 °C shaking at 220 rpm. 5 μl of each culture was used to inoculate 1 ml of 2 × TY containing 100 μg/ml ampicillin and either 0 μM or 36 μM tetracycline in 96-well deep well blocks. These colonies were then grown for 18 h shaking at 220 rpm at 37 °C and the cell density (OD_600_) of 200 μl was measured using the Tecan Safire II in a 96-well Nunclon flat-bottomed black plate (Sigma Aldrich), which was compared to wild type transporter as a positive control and vector containing no insert as a negative control. Expression of TetA(B) relied on leaky expression from the *lac*UV5 promoter in the absence of IPTG.

### 2.5 Liquid growth antibiotic resistance phenotypic assay of initial hits of TetA(B) site-directed point mutants

Colonies that were significantly less resistant to tetracycline as judged by relative optical density of cells grown in 36 μM tetracycline compared to cells grown in the absence of tetracycline were further examined over a broader range of tetracycline concentrations. 5 μl of tetracycline growth negative cultures from the 96-well plate containing only 100 μg/ml ampicillin in 2 × TY (16 g/l tryptone, 10 g/l yeast extract, 86 mM NaCl, pH 7.4) or from cultures grown for 18 h at 37 °C from freshly transformed JM109 cells was then added to 1 ml 2 × TY containing 100 μg/ml ampicillin containing 0 μM, 5 μM, 9 μM, 18 μM, 36 μM, 72 μM or 144 μM tetracycline in 96-deep well plates and grown at 220 rpm at 37 °C for 18 h. 200 μl samples were taken and the cell density (OD_600_) was measured using the Tecan Safire II and compared to JM109 cells transformed with wild type TetA(B)-Δ394–401-TEV-His10-eGFP in pBluescript SKII(+) as a positive control and pBluescript containing no insert as a negative control. Expression of TetA(B) relied on leaky expression from the *lac*UV5 promoter in the absence of IPTG.

### 2.6 Measurement of expression of TetA(B) mutants by in-gel fluorescence

1.5 ml of JM109 cells expressing transporter-eGFP fusions grown for 18 h at 37 °C at 220–250 rpm either in 24-well plates or in sterile glass tubes were harvested with a bench top Eppendorf 5424 centrifuge and resuspended in 100 μl deionised water. The cells were then sonicated, solubilised in SDS-PAGE buffer and analysed by SDS-PAGE on 4–20% Tris-Glycine gels (Invitrogen). Unstained gels were visualised on a Typhoon Trio (GE Healthcare) gel scanner with a green laser at 532 nm and expression levels of transporter-eGFP were compared to JM109 cells transformed with wild type transporter-eGFP fusions in pBluescript SKII(+) and pBluescript SKII(+) containing no insert.

### 2.7 Large-scale expression of TetA(B)His_10_ and TetA(B)-Δ394–401-Thrombin-His_12_

Expression of TetA(B)-His10 and TetA(B)-Δ394–401-Thrombin-His12 constructs were optimized in *E. coli* strain C43 [45,46]. Fresh transformations of each construct were made and plated on to 90 mm 2 × TY 1.5% (w/v) agar plates containing 100 μg/ml ampicillin. Colonies were picked into 10 ml of 2 × TY containing 100 μg/ml ampicillin and grown for 18 h in sterile test tubes at 37 °C and 250 rpm. 4 ml of this bacterial culture was used to inoculate 1 l of 2 × TY containing 100 μg/ml ampicillin in a 2 l conical flask, which was incubated at 37 °C, shaking at 220 rpm in an Infors HT Multitron standard shaker until the cell density reached an OD_600_ of 0.3–0.5. IPTG was added to a final concentration of 0.2 mM and the temperature was lowered to 27 °C for 4 h. Cells were harvested in 1 l centrifuge bottles (Beckman Coulter) at 3063 ×*g* for 15 min at 4 °C in a JLA-8.1000 rotor (Beckman Coulter) in Avanti J-26 XP centrifuge (Beckman Coulter) and resuspended in 20 ml per litre of cells of 20 mM Tris pH 7.4 with protease inhibitor tablets made up to manufacturer's specifications, which were then frozen at − 20 °C in 50 ml Falcon tubes.

### 2.8 Membrane isolation for transport assay and purification

Frozen cells were thawed and lysed by 3 passages using an Emulsiflex C3 high-pressure homogeniser (Avestin) at approximately 10,000 psi. Cell debris was removed by centrifugation in a Sorvall SS34 rotor at 27000 ×*g* for 45 min (4 °C) in a Sorvall Evolution RC centrifuge. The membrane fraction of this supernatant was collected by centrifugation in a Ti45 rotor (Beckman Coulter) for 120 min at 158,000 ×*g* (4 °C) in an Optima L-100 XP Ultracentrifuge (Beckman Coulter). The resulting supernatant was carefully removed and the pellet was resuspended in 20 mM Tris pH 7.4 with a homogenisation syringe (Samco), using 5 ml of buffer for every litre of starting culture. At this point aliquots of membranes could be frozen for transport assays, although usually the purification protocol was continued directly from resuspended membranes.

### 2.9 Ni^2 +^-affinity purification of TetA(B)∆394–401-Thrombin-His_12_ and TetA(B)His_10_ derivatives in LMNG for ITC

Membranes produced from 2 l of cells were solubilised in 1% LMNG (Affymetrix), 20 mM Tris pH 7.7, 20 mM MgCl_2_ and 20 mM NaCl and incubated for 2 h, rotating in 50 ml Falcon tubes at 4 °C. The insoluble material was removed by centrifugation from the solubilisate in a Ti70 rotor for 60 min at 311,000 ×*g* at 4 °C in an Optima L-100 XP Ultracentrifuge (Beckman) and the supernatant was transferred to 50 ml Falcon tubes. 1 ml bed volume of NiNTA-agarose beads (QIAGEN) was pre-equilibrated by centrifugation at 1500 ×*g* for 5 min at 4 °C in an Eppendorf 5430 R centrifuge to remove the supernatant, resuspension in 2 ml of 1% LMNG, 20 mM Tris pH 7.7, 20 mM MgCl_2_ and 20 mM NaCl, centrifugation for a further 5 min at 1500 ×*g* and removal of the supernatant. Imidazole to a concentration of 48 mM was added to the solubilisate, the pre-equilibrated beads were added and the mixtures were incubated rotating at 15 rpm for 2 h at 4 °C. The mixtures were then added to a Fisher brand filter column in a stepwise manner and washed with 40 ml of each of 52 mM and 64 mM imidazole containing 0.003% LMNG in the former, no LMNG in the latter and 20 mM Tris pH 7.7, 20 mM MgCl_2_ and 20 mM NaCl. TetA(B)His10 or TetA(B)∆394–401-Thrombin-His10 was eluted with 4 ml of 375 mM imidazole containing 20 mM Tris pH 7.7, 20 mM MgCl_2_ and 20 mM NaCl, dialysed for 14 h at 4 °C in 7 K cut-off SnakeSkin (Thermo Scientific) against 20 mM Tris pH 7.7, 20 mM MgCl_2_ and 20 mM NaCl and concentrated in a 4 ml Amicon Ultra Ultracel 50 K cut-off concentrator in Eppendorf 5430R centrifuge at 4 °C and 1500 ×*g* to a volume of approximately 250 μl. The protein concentration of this partially pure sample was then measured using an amido black assay [47].

### 2.10 Isothermal titration calorimetry

Isothermal titration calorimetry (ITC) was used to measure tetracycline binding to mutants and wild type TetA(B)-His_10_ and TetA(B)-Δ394–401-Thrombin-His_12_ that had been purified in LMNG (see Section 2.9). An ITC_200_ machine (Microcal) was used to measure the affinities of each mutant to tetracycline. The syringe and cell were first washed sequentially with 5% Decon 90, distilled water and buffer consisting of 20 mM Tris pH 7.7, 20 mM MgCl_2_ and 20 mM NaCl, and dried with an applied vacuum. The Microcal software provided with the ITC_200_ machine was used to identify initial experimental parameters using the settings “minimum protein” with an estimated affinity of 5 μM and enthalpy change of − 5 kJ. 280 μl of purified protein at 50 μM (diluted in the above dialysis buffer as required) was loaded in the cell and tetracycline made up at 750 μM in dialysis buffer was loaded in the syringe. Experiments were run at 20 °C with 20 injections: the first injection at 0.5 μl, followed by 19 injections at 2.0 μl. The apparatus was stirred at 1000 rpm and the delay between injections was set to 120 s. The software, Origin, was used to calculate the affinity and thermodynamic parameters for tetracycline binding to TetA(B) and the relevant mutants.

### 2.11 Tetracycline transport

Membranes were produced (Section 2.8) from cells expressing TetA(B)-His10 wild type, the mutants G44V, R70G and D285A and a negative control of cells identically induced with IPTG, but containing the pET21b vector and no insert. These membranes were then resuspended at 3.5 mg/ml total protein in 20 mM Tris pH 7.7, 20 mM MgCl_2_ and 20 mM NaCl, and 100 μl of membranes was used per transport assay. Transport was initiated with the addition of 10 μl tritiated tetracycline (95 μM cold and 5 μM ^3^H-tetracycline (American Radiolabelled Chemicals Inc, 0.07 mCi) in 20 mM Tris pH 7.7, 20 mM MgCl_2_ and 20 mM NaCl containing 10 mM ATP). Samples were made for each time point, vacuum filtered through a Multiscreen filter plate (Millipore 1.0 μm) prewashed with 0.1% polyethylenimine and washed three times with 200 μl of ice cold 20 mM Tris pH 7.7, 20 mM MgCl_2_ and 20 mM NaCl. The filter was then dried, scintillation fluid was added and radioactivity was measured for 60 s at each time point with a Tri-Carb 2910 TR. Each data point was performed in triplicate and fit in GraphPad Prism with a Michaelis–Menten non-linear fit.

## 3 Results

### 3.1 Analysis of TetA(B) mutants with deletions in an intracellular loop

**Fig. 1:**
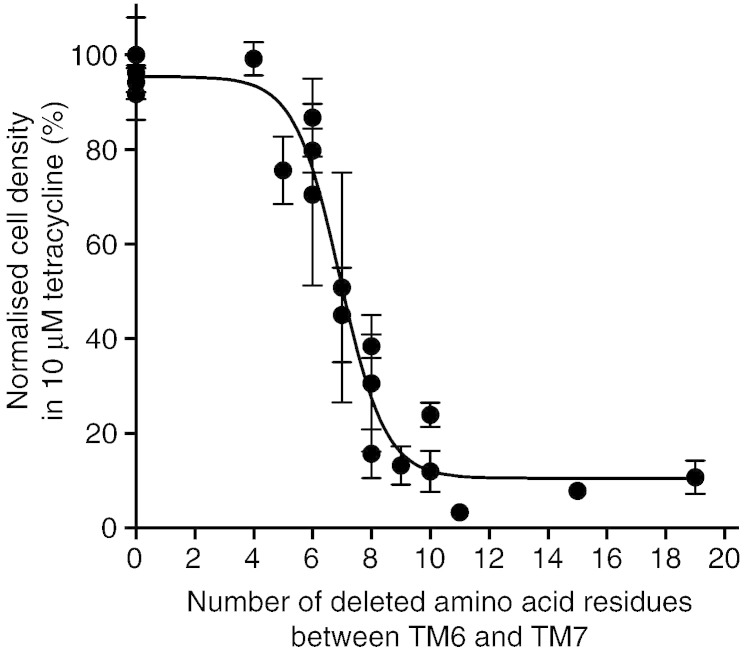
Tetracycline resistance phenotype of TetA(B) loop deletion mutants. Deletions in the loop between TM6 and TM7 of TetA(B) were analysed for their effect on transporter activity by measuring the tetracycline resistance of cells expressing the mutants. The OD_600_ of cultures grown in the presence of 10 μM tetracycline were compared to the OD_600_ of cultures grown in the absence of tetracycline, expressed as a percentage and plotted against the number of amino acid residues removed. The activity of each deletion mutant was measured in triplicate, with error bars representing the SEM and the data were then fit with a variable slope dose–response curve. Full data and sequences of the deletion mutants are in Supplementary Table 1.

MFS transporters have a long loop between transmembrane helix 6 (TM6) and TM7, which may be important to allow the conformation change between the outward-open and inward-open states. Shortening of this loop may therefore constrain the conformation of a given transporter. The loop between TM6 and TM7 in TetA(B), as predicted by the programme TMHMM [48,49], was predicted to be disordered by the RONN disorder prediction server [50]. Deletions were therefore made throughout this loop (Supplementary Table 1) ranging from 4 to 19 amino acid residues, avoiding residues Asp190, Glu192, Ser201 and Met210 that show reduced activity when mutated to Cys [33,51]. All deletions were made in full length TetA(B) fused at its C terminus to enhanced GFP (eGFP) in the pBluescript SKII (+) vector. The antibiotic resistance phenotype of these deletion mutants was then examined in *E. coli* strain JM109 by determining the MIC for tetracycline (Supplementary Table 1) and then measuring the optical density of cultures grown overnight in media containing 10 μM tetracycline (Fig. 1), relying on the basal activity of the *lac*UV5 promoter in the absence of IPTG to express the TetA(B) constructs. The tetracycline concentration of 10 μM was used for the latter experiment as this was the MIC for the negative control of JM109 cells not expressing TetA(B). In-gel fluorescence indicated that the expression level of each mutant was not significantly affected by the deletions (data not shown). It was possible to delete up to 6 amino acid residues and retain about 80% of the relative resistance phenotype in comparison to TetA(B) containing the full-length loop. However, removal of further amino acids reduced relative tetracycline resistance levels significantly and transporters that contained deletions of 9 or more amino acid residues conferred resistance to tetracycline to about 10% of the level observed with a full-length loop (Fig. 1), despite being expressed at similar levels. This also reduced the MIC of tetracycline to levels similar to JM109 cells not expressing TetA(B) (Supplementary Table 1).

### 3.2 Isolation of non-transporting TetA(B) mutants

Point mutations were made throughout TetA(B) from Asn2 to Ile379 using the Quickchange II methodology and expressed using the basal activity of the *lac*UV5 promoter in plasmid pBluescript (see Methods). The actual construct used, TetA(B)∆394–401-TEV-His10-eGFP, included a C-terminal truncation of 8 residues (∆394-401), a Tobacco Etch Virus (TEV) protease cleavage site, followed by 10 histidine residues (His10) and eGFP. The 8 C-terminal residues were predicted to be disordered by the RONN disorder prediction server [50] and were deleted to remove a proteolysis site that resulted in the expression of free eGFP in bacteria. The deletion did not affect the tetracycline resistance phenotype in comparison to the wild type transporter. Mutagenic oligonucleotide primers were designed to change each amino acid residue between positions 2 and 379. Each amino acid codon was changed to the sequence GBC (where B was a 1:1:1 ratio of the bases C, G and T) that introduced the codons GCC (Ala), GGC (Gly) or GTC (Val). Thus upon mutagenesis and transformation of *E. coli*, a mixture of mutants was obtained where each bacterial colony on a plate expressed TetA(B) that had been mutated at a single amino acid position, but which contained either a Val, Ala or Gly residue. It would be prohibitively expensive to sequence every mutant, so desirable mutants were first selected by measuring their ability to grow on media containing tetracycline. Up to 8 colonies were picked from each transformation to test for their resistance to tetracycline, apart from amino acid positions 113, 117, 137 and 228 where no colonies were produced and position 221, 233, 237, 262, 268, 272 and 281, where fewer than 5 colonies were produced. In total 3052 colonies were examined for their tetracycline resistance phenotype.

**Fig. 2:**
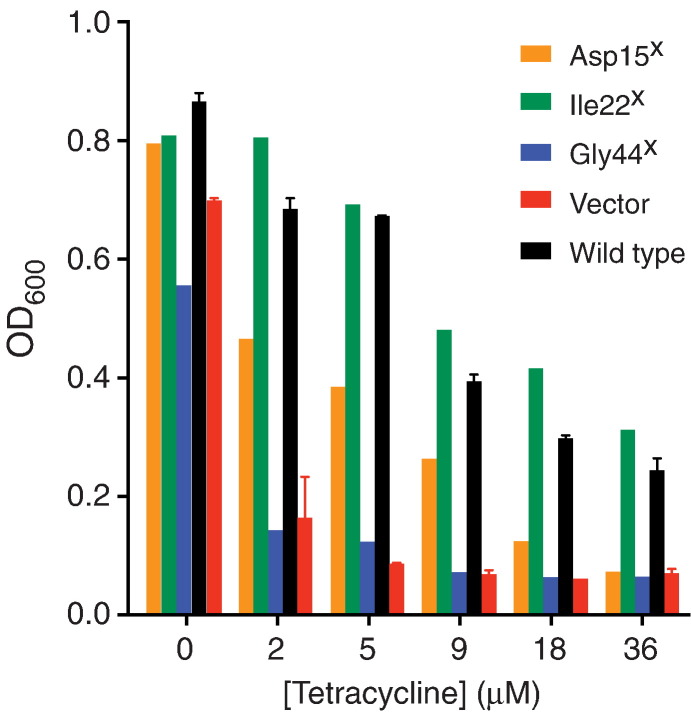
Selection of TetA(B) mutants that cannot confer tetracycline resistance to cells. Mutants that conferred significantly lowered cell density to *E. coli* after growth in the presence of 36 μM tetracycline were analysed over a range of tetracycline concentrations (0 μM–36 μM). Examples of mutants are shown compared to TetA(B)∆394-401-TEV-His10-eGFP (black, ‘wild type’) and a negative control of cells containing vector with no insert (red, ‘vector’). Several mutants were identified that, when re-tested, were able to confer full tetracycline resistance (e.g. Ile22^X^, green), whilst others showed significantly lowered, but measureable resistance to tetracycline (e.g. Asp15^X^, orange), and some showed growth indistinguishable with the negative control (e.g. Gly44^X,^ blue). The superscript ‘X’ implies that at this stage in the identification of the mutants, sequencing had not been performed, so the mutation of the defined amino acid residue could have been either to Val, Ala or Gly, or indeed another mutation could have been present elsewhere in the coding region. Positive and negative controls were performed in duplicate and error bars are shown (± SEM).

The ability of each TetA(B) mutant to transport tetracycline was assessed by measuring the ability of the mutant to confer tetracycline resistance *E. coli*. Interesting mutations that prevented tetracycline transport would thus manifest themselves as not growing on tetracycline-containing media. Each colony was therefore initially examined using a high-throughput single point antibiotic resistance phenotypic assay performed in 96-well plates. Growth was measured in media containing either no tetracycline or 36 μM tetracycline and was compared to cells either not expressing TetA(B) (negative control) or to cells expressing wild type TetA(B) (positive control). Of 3052 colonies tested, 519 showed growth to a significantly lower cell density than wild type i.e. the cell density attained in 36 μM tetracycline was less than 14% of the cell density in the absence of tetracycline (Supplementary Table 2). To exclude false positives, the 519 colonies were tested further using a multipoint tetracycline resistance assay (Fig. 2) where growth was measured in the presence of a range of tetracycline concentrations (0 μM, 5 μM, 9 μM, 18 μM, 36 μM, 72 μM and 144 μM). A total of 172 colonies were identified (Supplementary Table 2) that were consistently less resistant to tetracycline than wild type TetA(B) and were therefore examined for levels of TetA(B) expressed.

**Fig. 3:**
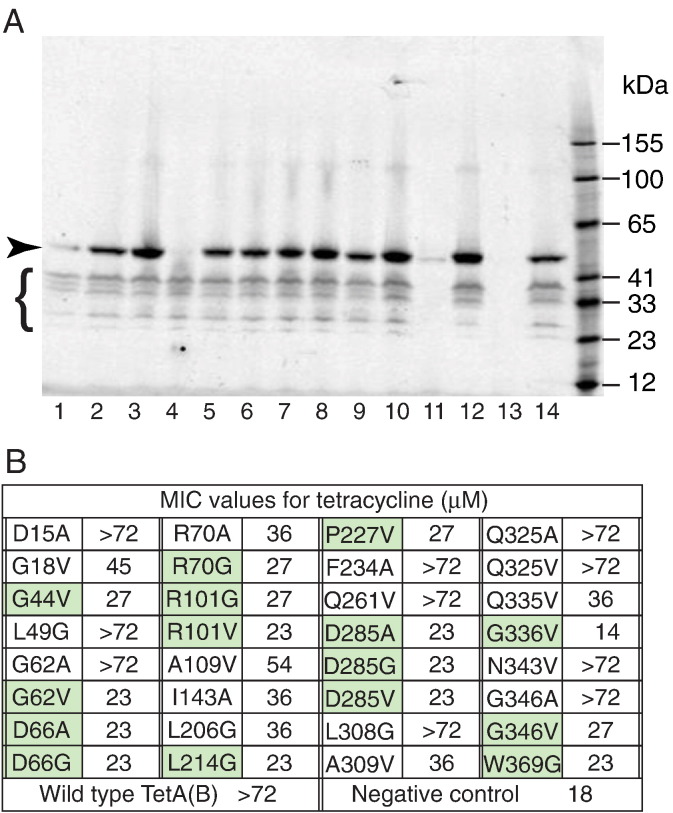
Measurement of TetA(B) mutant expression levels by in-gel fluorescence. A, The expression levels of TetA(B) mutants that did not confer tetracycline resistance to cells were examined by in-gel fluorescence. Lanes correspond to transport-compromised mutants: 1, T12^X^; 2, D15A; 3, G44V; 4, A58^X^; 5, G62V; 6, R70G; 7, R70A; 8, R101G; 9, R101V; 10, A109V; 11, G139^X^; 12, I143A; 13, vector-only negative control (pBluescript plasmid); 14, TetA(B)-∆394-401-TEV-His10-eGFP. After the in-gel fluorescence was performed, mutants that expressed to similar or higher levels than TetA(B)-∆394-401-TEV-His10-eGFP were sequenced to identify the mutation present. Those mutants that were expressed lower than TetA(B)-∆394-401-TEV-His10-eGFP were not sequenced, so the mutation(s) present were not defined, as indicated by the superscript ‘X’. The arrowhead identifies the full-length TetA(B)-∆394-401-TEV-His10-eGFP fusion protein and the curly bracket shows the position of degradation products. B, After the in-gel fluorescence analysis was completed and all the mutants were sequenced, 32 individual point mutations were identified that resulted in transport-defective TetA(B). All these mutants were re-tested for their ability to confer tetracycline resistance to cells and only those that showed a minimal inhibitory concentration (MIC) of tetracycline of ≤ 27 μM were characterised by ITC (green).

The expression levels of TetA(B) mutants was determined by in-gel fluorescence using the C-terminal eGFP fusion [52]. This was important because a significant reduction in the expression level of a TetA(B) mutant would give the same loss of tetracycline resistance phenotype to *E. coli* as a mutation that prevents conformation changes in the transporter. GFP remains fluorescent even in the presence of SDS [53], therefore SDS-PAGE was used to measure the expression levels of each mutant by in-gel fluorescence (Fig. 3). This was found to be more reliable than measuring whole-cell fluorescence. Of the 172 colonies tested, only 59 showed expression of a full-length mutant TetA(B)-eGFP fusion to similar levels as TetA(B)-∆394–401-TEV-His10-eGFP and hence only these were sequenced. Of the sequenced mutants, 33 contained a single point mutation, which represented 32 unique mutations. The minimal inhibitory concentration (MIC) for tetracycline was determined for these mutants (Fig. 3) and the fifteen mutants with the lowest MIC (≤ 27 μM tetracycline) were chosen for biophysical examination. The 15 mutations chosen were G44V, G62V, D66A, D66G, R70G, R101G, R101V, L214G, P227V, D285A, D285G, D285V, G336V, G346V and W369G.

### 3.3 Isothermal titration calorimetry

**Fig. 4:**
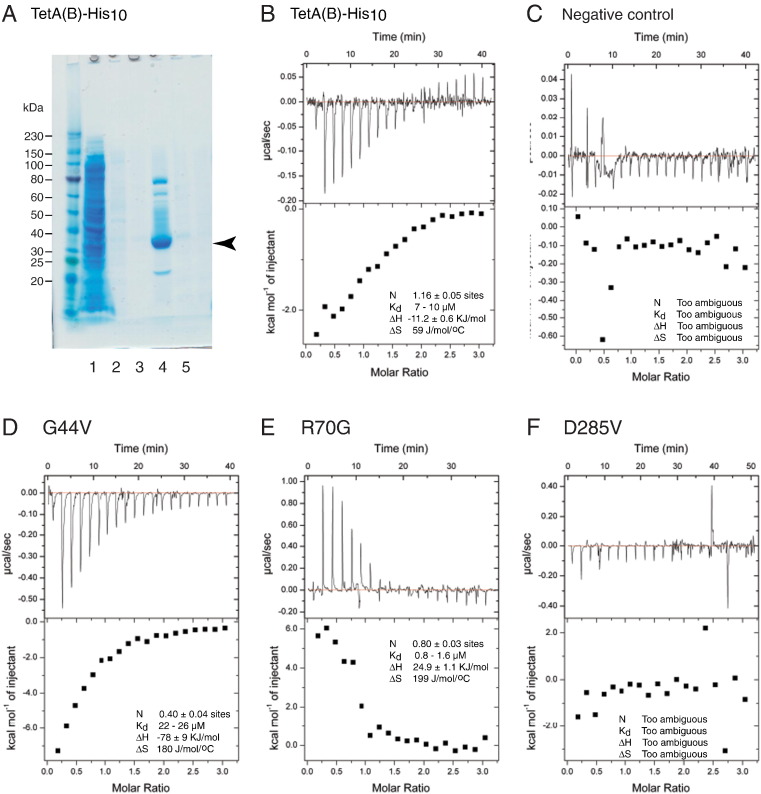
Isothermal titration calorimetry of TetA(B) non-transporting point mutants. A, TetA(B)-His_10_ was purified in the detergent LMNG by Ni^2 +^-affinity chromatography and the fractions were analysed on an SDS-PAGE gel; lane 1, flow through; lane 2, first wash; lane 3, second wash; lane 4, eluted sample. The eluted sample was concentrated and dialysed into a suitable buffer for ITC. Similar or better levels of purity were obtained with all the other TetA(B) constructs. B–F, examples of ITC data for TetA(B) mutants. Each experiment used a concentration of 50 μM TetA(B) mutant and injections of 0.5 μl–2 μl of 750 μM tetracycline. The parental TetA(B) construct used in all the experiments (except the negative control) was TetA(B)-His_10_. The negative control was tetracycline injected into detergent solution. B, positive control, TetA(B)-His_10_; C, negative control; D, G44V; E, R70G; F, D285V. Results for the number of binding sites (N), the affinity (K_d_), the change in enthalpy (ΔH) and the change in entropy (ΔS) upon tetracycline binding to each mutant was calculated from the data using the programme Origin. Where the data were inconsistent with tetracycline binding, we have written ‘Too ambiguous’. The data for ITC experiments performed on all mutants is summarised in Table 2, with the experimental data shown in Supplementary Figs. 1–5.

The affinity of TetA(B) for tetracycline was predicted to be in the μM range based on the K_m_ for transport [30,54] and therefore isothermal titration calorimetry (ITC) was used to measure the tetracycline binding affinities to the selected mutants. TetA(B)-His10 and TetA(B)-∆394–401-Thrombin-His10 were both purified in LMNG by Ni^2 +^-affinity chromatography and were of sufficient purity for ITC measurements to be performed. Both constructs resulted in a similar affinity (7.3 ± 0.5 μM and 10 ± 2 μM, respectively), showing that the C-terminal truncation had no significant effect on the binding of tetracycline to TetA(B) (Table 1). Therefore, the 15 point mutations were transferred to either TetA(B)-His_10_ (G44V, G62V, R70G, R101G, R101V, P227V, D285A, D285G, D285V, G336V and G346V) or TetA(B)∆394–401-Thrombin-His_12_ (loop deletions and D66A, D66G, L214G and W369G) for purification and ITC measurements (Fig. 4, Table 1 and Supplementary Figs. 1–5).

**Fig. 5:**
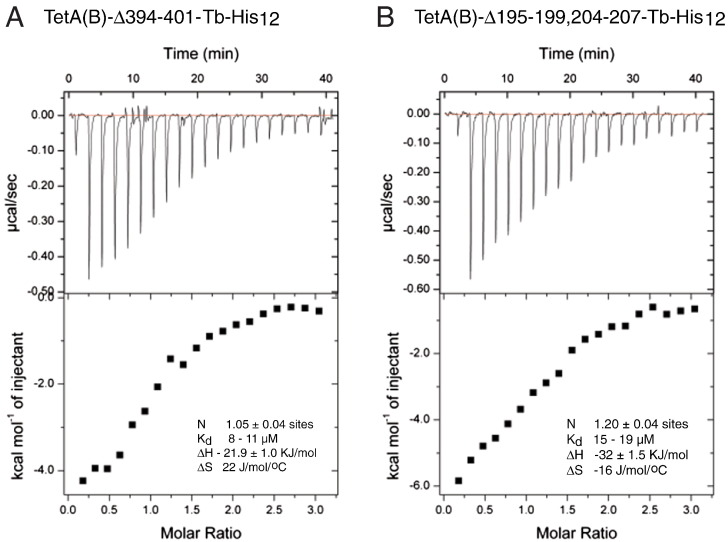
Isothermal titration calorimetry of TetA(B) loop deletion mutants that are transport-defective. Loop deletion mutants were constructed in TetA(B)-∆394–401-Tb-His12; the C-terminal deletion (∆394–401) is not included in their names for clarity. A, positive control TetA(B)-∆394–401-Tb-His_12_; B, TetA(B)-∆195–199,204–207-Tb-His_12_. The data for ITC experiments performed on other loop deletion mutants is summarised in Table 2, with the experimental data shown in Supplementary Fig. 5.

Of the 15 mutants that resulted in a tetracycline-sensitive phenotype to *E. coli*, only 3 mutants (G44V, R70G and G346V) could be readily purified and reproducibly bound tetracycline (Table 1). The mutant R70G was particularly interesting because it bound tetracycline with a K_d_ of 2.1 ± 0.9 μM, which was 3.5-fold higher affinity compared to TetA(B)-His_10_, whereas tetracycline bound to the mutants G44V and G346V (K_d_ 28 ± 4 μM and 36 ± 16 μM, respectively) between 4-fold and 5-fold weaker than the parental transporter. Of the remaining mutants, G336V was not overexpressed and therefore could not be purified in sufficient amounts for ITC, L214G and P227V gave ambiguous results and the remainder showed no appreciable binding of tetracycline (G62V, D66A, D66G, R101G, R101V, D285A, D285G, D285V and W369G). Two loop deletion mutants, TetA(B)-Δ195–199,203–207 (9 amino acid residues deleted) and TetA(B)-Δ182–187,195–199 (11 amino acid residues deleted), were also examined for tetracycline binding (Fig. 5). The deletion of 9 amino acid residues resulted in only a minor change in tetracycline affinity relative to the parental transporter (K_d_ 17.5 ± 0.5 μM compared to 10 ± 2 μM). However, deletion of 11 amino acid residues resulted in no measureable tetracycline binding.

### 3.4 Tetracycline transport of selected mutants

**Fig. 6:**
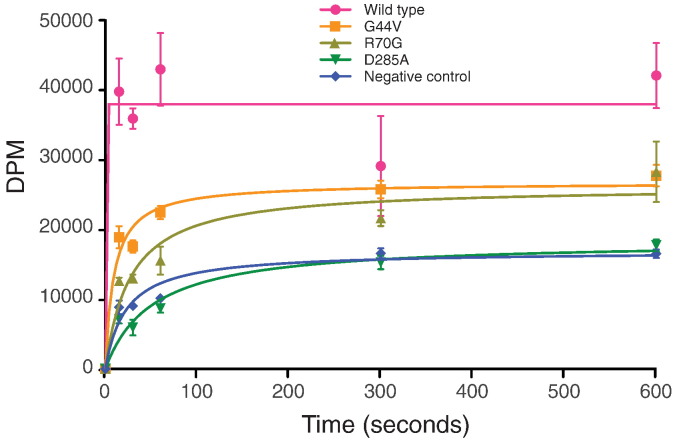
Tetracycline transport in inverted membrane vesicles. Inverted vesicles were prepared from whole *E. coli* cells expressing each TetA(B) mutant identified that did not confer tetracycline resistance. The transport of ^3^H-tetracycline was measured in the presence of ATP: pink circles, wild type TetA(B); orange squares, TetA(B)-G44V; khaki green triangles, TetA(B)-R70G; green inverted triangles, TetA(B)-D285A; blue diamonds, negative control (no TetA(B) expressed).

To further characterise selected mutants of TetA(B), H^+^-linked transport of ^3^H-tetracycline was measured in membrane vesicles prepared from cells expressing the transporters. The ^3^H-tetracycline transport rate of TetA(B)-His_10_ was compared with the low affinity mutant G44V, the high affinity mutant R70G, the non-tetracycline-binding mutant D285A and a negative control of membranes containing no over-expressed TetA(B). ^3^H-tetracycline transport by membranes containing the mutant D285A was indistinguishable from the negative control (Fig. 6). In contrast, ^3^H-tetracycline transport was observed in membranes containing the mutants G44V and R70G that were still able to bind tetracycline, although transport was considerably diminished compared to TetA(B)-His_10_ (Fig. 6).

## 4 Discussion

Crystallisation and structure determination of proton-linked MFS transporters has made great progress over the last 15 years since the first structure, LacY, was determined [4–29]. However, the number of structures that have been solved is still a tiny fraction of unique transporters found in nature. One possibility to account for the difficulty in crystallising MFS transporters is that there is considerable conformational heterogeneity of the purified transporter, particularly as one of the substrates, a H^+^, can never be removed from solution whilst maintaining the structure of the transporter. Thus there is little impediment to transitions between the outward-open and inward-open states, which inevitably makes the formation of well-diffracting crystals problematic. Therefore the isolation of mutants of a transporter that maintain the structure of the wild type protein and yet are conformationally restrained would greatly facilitate structure determination. Here we showed that two systematic methodologies generated mutants that were significantly impaired in their ability to transport tetracycline and yet some of these mutants could still bind tetracycline.

Deletions in the loop between TM6 and TM7 have previously been made in LacY, the most well-studied member of the MFS family. It was observed that deletion of five residues resulted in a transporter that was essentially indistinguishable from wild type, whereas deletion of 12 or 20 residues resulted in a transporter that did not transport the substrate lactose [55]. Deletions in the analogous loop in TetA(B) gave similar results, with the removal of 9 or more amino acid residues resulting in TetA(B) mutants that did not confer tetracycline resistance to cells. Interestingly, removal of 9 amino acid residues still resulted in a TetA(B) mutant that could bind tetracycline with similar affinity to the wild type transporter, suggesting that it could be conformationally restrained and is thus a good candidate for structural studies.

**Fig. 7:**
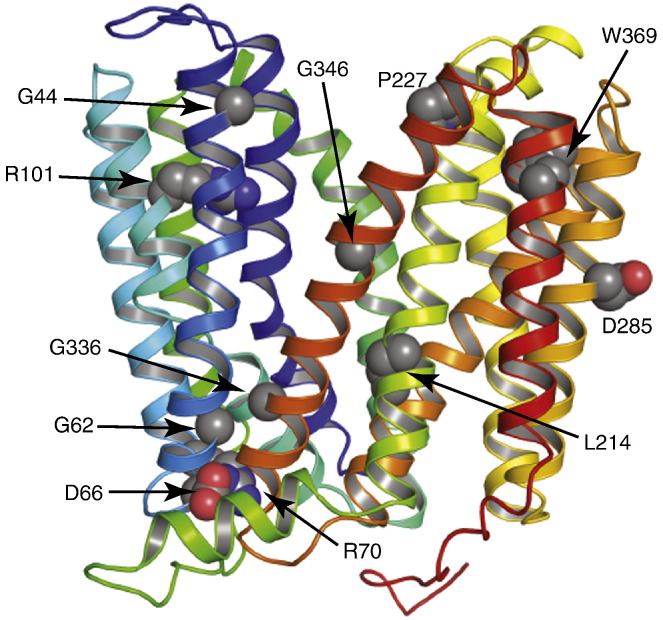
Predicted positions of non-transporting mutants based on a model of the TetA(B) homolog YajR. A model of TetA(B) was built from YajR (PDB code 3wdo) [21], using SWISS-MODEL [60] and an alignment with AlignMe [61]. The positions of non-transporting mutants are shown in grey and labelled. The model is viewed in the membrane plane.

An alternative strategy to identify potentially conformationally-restrained transporters was to isolate point mutations in TetA(B) that abolished the tetracycline-resistant phenotype conferred by the wild type transporter on *E. coli*. Fifteen such mutants of TetA(B) were identified at 11 amino acid residues throughout the transporter with similar expression levels to the wild type transporter. Interestingly, of these 11 amino acid residues, 8 gave a similar reduced transport phenotype when the residues were mutated to Cys [33–39] (amino acid residues G44, G62, D66, R70, R101, D285, G336, G346), whereas 3 did not (amino acid residues L214, P227, W369). It is striking that the majority of these mutations are either of a Gly residue or a charged residue, suggesting that scanning mutagenesis to identify transport-defective mutations in other transporters could be streamlined by considering only these residues. Three main causes for the effects of the mutations can be envisaged: (1) the mutation alters an amino acid residue that makes direct contact to tetracycline, thus significantly lowering its affinity and hence abolishing substrate binding; (2) the mutation alters an amino acid residue that is at the interface between transmembrane helices, thus increasing the kinetic barriers between different conformational states and hence abolishing transport; (3) the mutation perturbs the folding of the transporter so that the mutant does not attain a correctly folded state. Ideally, each mutant should be characterised to define the most likely effect it had on TetA(B) folding and activity, thus identifying those mutants most likely to improve the probability of obtaining well-diffracting crystals. To facilitate this, the positions of the mutations were placed on a model of TetA(B) made simply from threading the amino acid sequence on to the structure of YajR [21] (Fig. 7). The structures of MFS transporters [4–29] show that substrates bind invariably in the V-shaped cleft between the N-terminal and C-terminal halves of the protein, so if this also applies to TetA(B), then the model suggests that none of the mutations would have had a significant direct impact upon the affinity of tetracycline binding.

To differentiate between whether a given mutation results in a misfolded transporter or a correctly folded transporter, the ability of the mutant to bind tetracycline was measured by ITC. Three mutants did indeed bind tetracycline, G44V, R70G and G346V, thus highlighting these as useful candidates for structural studies. Of the remaining mutants, it is unclear from the data whether they are correctly folded or not. The absence of tetracycline binding does not prove that a mutant is misfolded, because it is possible that the mutant is in an occluded state that prevents substrate binding. In fact, circumstantial evidence supports the possibility that these mutants may indeed be in a biologically relevant conformation given that all the mutants (except for G336V that did not overexpress) could be purified using the very mild detergent LMNG. Misfolded membrane proteins have a strong propensity to aggregate in the membrane that usually prevents their solubilisation by mild detergents such as dodecylmaltoside [56] and also they tend to be more prone to aggregation during purification. However, there is no easy way to ascertain whether these mutants are indeed folded, without extensive biophysical data, and the best way to prove this is to see whether they crystallise (work in progress).

One cluster of mutants, G62A, D66A, R70G and G336V, were predicted to be close together in 3-dimensional space in the outward-open model (Fig. 7). The residues Gly62, Asp66 and Arg70 and the region between TM2 and TM3, is highly conserved and has been implicated in gating [57]. It is interesting that, although Asp66 and Arg70 are both predicted to be involved in gating [57], the effects of mutating these residues were different and the roles of Asp66 and Arg70 are not analogous. The TetA(B) mutants D66A and D66G were transport-defective and could not bind tetracycline whereas the mutant R70G bound tetracycline with high affinity despite being transport-defective. The TetA(B) mutant R70G bound tetracycline with a significantly higher affinity than wild type (2.1 μM compared to 7.3 μM) and, in addition, showed markedly different thermodynamic parameters for tetracycline binding (Table 1). Interestingly, analogous results were observed for the conformationally stabilised C154G mutant of the lactose permease, LacY [58], which was subsequently crystallised and gave the first structure of an MFS transporter. Similar parallels are observed between the G44V mutant in TetA(B) and the G46W in LacY. TetA(B)-G44V was transport-defective and yet still bound tetracycline, although with a 4-fold reduced affinity compared to the wild type transporter. LacY-G46W was unable to transport lactose, but could still bind it, and was instrumental in crystallising an alternative LacY conformation [9,59]. This striking parallel suggests that a more rational approach to conformational stabilisation of MFS transporters through rational mutagenesis may be possible, due to the conserved mechanism of transport within this family. The work presented here is a first step towards this goal and suggests that the recent successes in developing generic technologies for the crystallisation of GPCRs could be mimicked with MFS transporters.

## Conflict of interest

None.

## Figures and Tables

**Table 1:** ITC data for tetracycline binding to purified transport-defective TetA(B) mutants.

Construct	Mutant	Number of independent experiments	Affinity K_d_ (μM)	Enthalpy ΔH (kJ/mol)	EntropyΔS (J/mol)	Tetracycline bound to TetA(B) N (mol/mol)
TetA(B)-His10		3	7.3 ± 0.5	-11.6 ± 1.0	59 ± 3	1.3 ± 0.2
	G44V	2	28 ± 4	-75.5 ± 2.5	170 ± 10	0.4 ± 0.2
	G62V	2	No significant binding			
	R70G	2	2.1 ± 0.9	24 ± 1	189 ± 10	1.14 ± 0.34
	R101G	3	No significant binding			
	R101V	2	No significant binding			
	P227V	2	33 ± 12	-14 ± 5	39	0.6 ± 0.2
			No significant binding			
	D285A	2	No significant binding			
	D285G	2	No significant binding			
	D285V	2	No significant binding			
	G336V	No expression				
	G346V	2	36 ± 16	-27 ± 9	34 ± 6	1.3 ± 0.1
TetA(B)-Δ394-401-Tb-His12		2	10 ± 2	-22 ± 1	20.5 ± 1.5	1.1 ± 0.1
	D66A	2	No significant binding			
	D66G	2	No significant binding			
	L214G	3	31 ± 12	-14 ± 12	39	0.3 ± 0.2
			No significant binding (n = 2)			
	W369G	2	No significant binding			
	Δ195–199, 203–207	2	17.5 ± 0.5	-29 ± 3	-7 ± 9	1.1 ± 0.1
	Δ182–187, 195–199	2	No significant binding			
