# Supplementary material for: Isolation and characterisation of transport-defective substrate-binding mutants of the tetracycline antiporter TetA(B)
Source: Biochim Biophys Acta. 2015 Oct;1848(10):2261–70. doi: 10.1016/j.bbamem.2015.06.026 (PMC4579554; doi:10.1016/j.bbamem.2015.06.026)
Supplement: Supplementary file 1 — Supplementary material. [file mmc1.pdf]

| Deletion | Sequence | Normalized growth (Mean) | MIC of tetra-cycline (μM) | Number of amino acids removed |
|---|---|---|---|---|
| Wild type | VVMFWFRETKNTRDNTDTEVGVETQSNSVYITLFKTMPILLIIY | 100% | >50 | 0 |
| Δ204-207 | VVMFWFRETKNTRDNTDTEVGVETQSNSVY----KTMPILLIIY | 104% | >50 | 4 |
| Δ195-199 | VVMFWFRETKNTRDNTDTEVG-----NSVYITLFKTMPILLIIY | 79% | >50 | 5 |
| Δ182-187 | VVMFWFR------DNTDTEVGVETQSNSVYITLFKTMPILLIIY | 83% | >50 | 6 |
| Δ195-200 | VVMFWFRETKNTRDNTDTEVG------SVYITLFKTMPILLIIY | 91% | >50 | 6 |
| Δ202-207 | VVMFWFRETKNTRDNTDTEVGVETQSNS------KTMPILLIIY | 73% | >50 | 6 |
| Δ193-199 | VVMFWFRETKNTRDNTDTE-------NSVYITLFKTMPILLIIY | 53% | 50 | 7 |
| Δ201-207 | VVMFWFRETKNTRDNTDTEVGVETQSN-------KTMPILLIIY | 46% | 50 | 7 |
| Δ180-187 | VVMFW--------DNTDTEVGVETQSNSVYITLFKTMPILLIIY | 32% | 50 | 8 |
| Δ182-189 | VVMFWFR--------TDTEVGVETQSNSVYITLFKTMPILLIIY | 15% | 50 | 8 |
| Δ200-207 | VVMFWFRETKNTRDNTDTEVGVETQS--------KTMPILLIIY | 39% | >50 | 8 |
| Δ195-199 Δ204-207 | VVMFWFRETKNTRDNTDTEVG-----NSVY----KTMPILLIIY | 13% | 20 | 9 |
| Δ198-207 | VVMFWFRETKNTRDNTDTEVGVET----------KTMPILLIIY | 24% | 20 | 10 |
| Δ182-187 Δ204-207 | VVMFWFR------DNTDTEVGVETQSNSVY----KTMPILLIIY | 11% | 10 | 10 |
| Δ182-187 Δ195-199 | VVMFWFR------DNTDTEVG-----NSVYITLFKTMPILLIIY | 2% | 20 | 11 |
| Δ182-187 Δ200-208 | VVMFWFR------DNTDTEVGVETQ----------TMPILLIIY | 8% | 20 | 15 |
| Δ182-187 Δ196-207 | VVMFWFR------DNTDTEVGV------------KTMPILLIIY | 10% | 20 | 19 |
| Negative control | Vector with no insert | 0% | 10 | n/a |

Supplementary Table 1. Activity of deletion mutants constructed between TM6 and TM7. The amino acid sequence of each mutant is shown, with the underlined regions representing amino acid residues predicted to be in the cytoplasmic ends of TM6 and TM7. The activity data are depicted graphically in Figure 1. Mutants with deletions of 9 and 11 amino acid residues were purified and the binding analysed by ITC (Fig. 5).

**Supplementary Table 2.** Summary of all VAG scan data in TetA(B) from Asn2 to Ile379. Columns (from left to right) show the following data: the amino acid residue in TetA(B) where the mutation was introduced; the predicted transmembrane helix; the number of colonies picked from the transformation of mutagenic PCR; the number of colonies with significantly reduced resistance to tetracycline, determined by the ratio of $OD_{600}$ grown at 0 µM and 16 µM tetracycline; the number of colonies tested that showed a consistent loss of tetracycline resistance phenotype; the sequence of the colonies, where available. Residues 380-401 were not mutated.

| Amino acid | TM prediction (TMHMM) | Number of colonies assayed for growth and whole-cell fluorescence | Initial hits ($OD_{600}$ 16 µg/ml/0 µg.ml tetracycline <0.14) | Reproducibly not tetracycline resistant | In-gel fluorescence at TetA(B)-TEV-His10-eGFP size | Sequence of transport-compromised mutants |
|---|---|---|---|---|---|---|
| N2 | | 16 | 6 | 0 | - | |
| S3 | | 8 | 0 | 0 | - | |
| S4 | | 8 | 0 | 0 | - | |
| T5 | | 8 | 0 | 0 | - | |
| K6 | | 8 | 1 | 0 | - | |
| I7 | 1 | 8 | 0 | 0 | - | |
| A8 | 1 | 8 | 0 | 0 | - | |
| L9 | 1 | 8 | 0 | 0 | - | |
| V10 | 1 | 8 | 0 | 0 | - | |
| I11 | 1 | 8 | 0 | 0 | - | |
| T12 | 1 | 8 | 1 | 1 | Yes | T12A |
| L13 | 1 | 8 | 0 | 0 | - | |
| L14 | 1 | 8 | 0 | 0 | - | |
| D15 | 1 | 8 | 5 | 1 | Yes | D15A |
| A16 | 1 | 8 | 2 | 1 | No | |
| M17 | 1 | 8 | 1 | 1 | No | |
| G18 | 1 | 8 | 2 | 1 | Yes | G18V |
| I19 | 1 | 8 | 1 | 0 | - | |
| G20 | 1 | 8 | 1 | 0 | - | |
| L21 | 1 | 8 | 1 | 1 | Yes | Not correct vector |
| I22 | 1 | 8 | 2 | 1 | No | |
| M23 | 1 | 8 | 0 | 0 | - | |
| P24 | 1 | 8 | 2 | 1 | No | |
| V25 | 1 | 8 | 0 | 0 | - | |
| L26 | 1 | 8 | 1 | 1 | Yes | Not correct vector |
| P27 | 1 | 8 | 2 | 1 | No | |
| T28 | 1 | 8 | 0 | 0 | - | |
| L29 | 1 | 8 | 8 | 0 | - | |
| L30 | | 8 | 1 | 1 | No | |
| R31 | | 8 | 3 | 0 | - | |
| E32 | | 9 | 2 | 0 | - | |
| F33 | | 8 | 1 | 0 | - | |
| I34 | | 8 | 0 | 0 | - | |
| A35 | | 8 | 1 | 0 | - | |
| S36 | | 8 | 1 | 0 | - | |

| Amino acid | TM prediction (TMHMM) | Number of colonies assayed for growth and whole-cell fluorescence | Initial hits ($OD_{600}$ 16 µg/ml/0 µg.ml tetracycline <0.14) | Reproducibly not tetracycline resistant | In-gel fluorescence at TetA(B)-TEV-His10-eGFP size | Sequence of transport-compromised mutants |
|---|---|---|---|---|---|---|
| E37 | | 8 | 0 | 0 | - | |
| D38 | | 8 | 0 | 0 | - | |
| I39 | 2 | 8 | 0 | 0 | - | |
| A40 | 2 | 8 | 0 | 0 | - | |
| N41 | 2 | 8 | 4 | 1 | No | |
| H42 | 2 | 8 | 0 | 0 | - | |
| F43 | 2 | 8 | 0 | 0 | - | |
| G44 | 2 | 8 | 3 | 2 | Yes | G44V |
| V45 | 2 | 8 | 0 | 0 | - | |
| L46 | 2 | 8 | 0 | 0 | - | |
| L47 | 2 | 8 | 0 | 0 | - | |
| A48 | 2 | 8 | 0 | 0 | - | |
| L49 | 2 | 8 | 0 | 0 | - | |
| Y50 | 2 | 8 | 0 | 0 | - | |
| A51 | 2 | 8 | 1 | 1 | Yes | P59R |
| L52 | 2 | 8 | 0 | 0 | - | |
| M53 | 2 | 8 | 1 | 0 | - | |
| Q54 | 2 | 8 | 0 | 0 | - | |
| V55 | 2 | 8 | 0 | 0 | - | |
| I56 | 2 | 8 | 2 | 1 | - | |
| F57 | 2 | 8 | 1 | 0 | - | |
| A58 | 2 | 8 | 3 | 1 | No | |
| P59 | 2 | 8 | 2 | 1 | No | |
| W60 | 2 | 8 | 0 | 0 | - | |
| L61 | 2 | 8 | 1 | 1 | No | |
| G62 | | 8 | 4 | 2 | Yes x2 | G62A and G62V |
| K63 | | 8 | 3 | 1 | Yes | Not correct vector |
| M64 | | 8 | 0 | 0 | - | |
| S65 | | 8 | 1 | 0 | - | |
| D66 | | 8 | 7 | 3 | Yes x2 | D66A and D66G |
| R67 | | 8 | 0 | 0 | - | |
| F68 | | 8 | 0 | 0 | - | |
| G69 | | 8 | 4 | 0 | - | |
| R70 | | 8 | 7 | 4 | Yes x4 | R70G and R70A |
| R71 | | 8 | 0 | 0 | - | |
| P72 | | 8 | 0 | 0 | - | |
| V73 | 3 | 8 | 0 | 0 | - | |
| L74 | 3 | 8 | 0 | 0 | - | |
| L75 | 3 | 8 | 0 | 0 | - | |
| L76 | 3 | 8 | 2 | 0 | - | |
| S77 | 3 | 8 | 0 | 0 | - | |
| L78 | 3 | 8 | 0 | 0 | - | |
| I79 | 3 | 8 | 1 | 1 | No | |

| Amino acid | TM prediction (TMHMM) | Number of colonies assayed for growth and whole-cell fluorescence | Initial hits ($OD_{600}$ 16 µg/ml/0 µg.ml tetracycline <0.14) | Reproducibly not tetracycline resistant | In-gel fluorescence at TetA(B)-TEV-His10-eGFP size | Sequence of transport-compromised mutants |
|---|---|---|---|---|---|---|
| G80 | 3 | 8 | 0 | 0 | - | |
| A81 | 3 | 8 | 0 | 0 | - | |
| S82 | 3 | 8 | 1 | 0 | - | |
| L83 | 3 | 8 | 1 | 0 | - | |
| D84 | 3 | 8 | 4 | 0 | - | |
| Y85 | 3 | 8 | 0 | 0 | - | |
| L86 | 3 | 8 | 1 | 1 | No | |
| L87 | 3 | 8 | 0 | 0 | - | |
| L88 | 3 | 8 | 1 | 0 | - | |
| A89 | 3 | 8 | 0 | 0 | - | |
| F90 | 3 | 8 | 2 | 1 | No | |
| S91 | 3 | 8 | 1 | 0 | - | |
| S92 | 3 | 8 | 0 | 0 | - | |
| A93 | 3 | 8 | 0 | 0 | - | |
| L94 | 3 | 8 | 0 | 0 | - | |
| W95 | 3 | 8 | 0 | 0 | - | |
| M96 | | 8 | 3 | 0 | - | |
| L97 | | 8 | 3 | 1 | Yes | A81V + L97G |
| Y98 | | 8 | 0 | 0 | - | |
| L99 | 4 | 8 | 0 | 0 | - | |
| G100 | 4 | 8 | 4 | 0 | - | |
| R101 | 4 | 8 | 1 | 1 | Yes | R101G |
| L102 | 4 | 8 | 0 | 0 | - | |
| L103 | 4 | 8 | 0 | 0 | - | |
| S104 | 4 | 8 | 0 | 0 | - | |
| G105 | 4 | 8 | 1 | 0 | - | |
| I106 | 4 | 8 | 1 | 1 | No | |
| T107 | 4 | 8 | 2 | 0 | - | |
| G108 | 4 | 8 | 0 | 0 | - | |
| A109 | 4 | 8 | 3 | 2 | Yes | A109V |
| T110 | 4 | 8 | 4 | 1 | Yes | R101V +T110G |
| G111 | 4 | 8 | 3 | 1 | Yes | R101V |
| A112 | 4 | 8 | 0 | 0 | - | |
| V113 | 4 | 0 | 0 | 0 | - | |
| A114 | 4 | 8 | 0 | 0 | - | |
| A115 | 4 | 8 | 0 | 0 | - | |
| S116 | 4 | 8 | 0 | 0 | - | |
| V117 | 4 | 0 | 0 | 0 | - | |
| I118 | 4 | 8 | 2 | 1 | Yes | A112V + A119V |
| A119 | | 8 | 4 | 0 | - | |
| D120 | | 8 | 3 | 2 | Yes | D120A |
| T121 | | 8 | 0 | 0 | - | |
| T122 | | 8 | 4 | 0 | - | |
| S123 | | 8 | 3 | 0 | - | |

| Amino acid | TM prediction (TMHMM) | Number of colonies assayed for growth and whole-cell fluorescence | Initial hits ($OD_{600}$ 16 µg/ml/0 µg.ml tetracycline <0.14) | Reproducibly not tetracycline resistant | In-gel fluorescence at TetA(B)-TEV-His10-eGFP size | Sequence of transport-compromised mutants |
|---|---|---|---|---|---|---|
| A124 | | 8 | 0 | 0 | - | |
| S125 | | 16 | 5 | 3 | Yes x3 | Multiple mutations: at 109, 116, 125 and 135/151/164 |
| Q126 | | 8 | 0 | 0 | - | |
| R127 | | 8 | 0 | 0 | - | |
| V128 | | 8 | 1 | 0 | - | |
| K129 | | 8 | 0 | 0 | - | |
| W130 | 5 | 8 | 2 | 0 | - | |
| F131 | 5 | 8 | 0 | 0 | - | |
| G132 | 5 | 8 | 0 | 0 | - | |
| W133 | 5 | 8 | 0 | 0 | - | |
| L134 | 5 | 8 | 0 | 0 | - | |
| G135 | 5 | 8 | 3 | 2 | Yes | R127G + I143V |
| A136 | 5 | 8 | 2 | 2 | No | |
| S137 | 5 | 0 | 0 | 0 | - | |
| F138 | 5 | 8 | 0 | 0 | - | |
| G139 | 5 | 8 | 3 | 2 | No | |
| L140 | 5 | 8 | 0 | 0 | - | |
| G141 | 5 | 8 | 2 | 0 | - | |
| L142 | 5 | 8 | 1 | 0 | - | |
| I143 | 5 | 8 | 1 | 1 | Yes | I143A |
| A144 | 5 | 8 | 0 | 0 | - | |
| G145 | 5 | 8 | 1 | 0 | - | |
| P146 | 5 | 8 | 0 | 0 | - | |
| I147 | 5 | 8 | 0 | 0 | - | |
| I148 | 5 | 8 | 0 | 0 | - | |
| G149 | 5 | 8 | 3 | 0 | - | |
| G150 | 5 | 8 | 1 | 0 | - | |
| F151 | 5 | 8 | 0 | 0 | - | |
| A152 | 5 | 8 | 0 | 0 | - | |
| G153 | | 8 | 0 | 0 | - | |
| E154 | | 8 | 0 | 0 | - | |
| I155 | | 8 | 1 | 0 | - | |
| S156 | | 8 | 0 | 0 | - | |
| P157 | | 8 | 0 | 0 | - | |
| H158 | | 8 | 0 | 0 | - | |
| S159 | | 8 | 0 | 0 | - | |
| P160 | | 8 | 0 | 0 | - | |
| F161 | | 8 | 0 | 0 | - | |
| F162 | 6 | 8 | 0 | 0 | - | |
| I163 | 6 | 8 | 0 | 0 | - | |
| A164 | 6 | 8 | 5 | 0 | - | |
| A165 | 6 | 8 | 3 | 0 | - | |
| L166 | 6 | 8 | 0 | 0 | - | |

| Amino acid | TM prediction (TMHMM) | Number of colonies assayed for growth and whole-cell fluorescence | Initial hits ($OD_{600}$ 16 µg/ml/0 µg.ml tetracycline <0.14) | Reproducibly not tetracycline resistant | In-gel fluorescence at TetA(B)-TEV-His10-eGFP size | Sequence of transport-compromised mutants |
|---|---|---|---|---|---|---|
| L167 | 6 | 8 | 0 | 0 | - | |
| N168 | 6 | 8 | 1 | 0 | - | |
| I169 | 6 | 8 | 0 | 0 | - | |
| V170 | 6 | 8 | 1 | 0 | - | |
| T171 | 6 | 8 | 1 | 0 | - | |
| F172 | 6 | 8 | 1 | 0 | - | |
| L173 | 6 | 8 | 3 | 0 | - | |
| V174 | 6 | 8 | 0 | 0 | - | |
| V175 | 6 | 8 | 0 | 0 | - | |
| M176 | 6 | 8 | 0 | 0 | - | |
| F177 | 6 | 8 | 0 | 0 | - | |
| W178 | 6 | 7 | 0 | 0 | - | |
| F179 | 6 | 7 | 0 | 0 | - | |
| R180 | | 8 | 0 | 0 | - | |
| E181 | | 8 | 0 | 0 | - | |
| T182 | | 8 | 1 | 0 | - | |
| K183 | | 8 | 1 | 0 | - | |
| N184 | | 8 | 1 | 0 | - | |
| T185 | | 8 | 0 | 0 | - | |
| R186 | | 8 | 0 | 0 | - | |
| D187 | | 5 | 4 | 0 | - | |
| N188 | | 8 | 0 | 0 | - | |
| T189 | | 8 | 0 | 0 | - | |
| D190 | | 16 | 2 | 0 | - | |
| T191 | | 8 | 0 | 0 | - | |
| E192 | | 8 | 1 | 0 | - | |
| V193 | | 8 | 0 | 0 | - | |
| G194 | | 8 | 0 | 0 | - | |
| V195 | | 8 | 0 | 0 | - | |
| E196 | | 16 | 6 | 0 | - | |
| T197 | | 8 | 0 | 0 | - | |
| Q198 | | 8 | 0 | 0 | - | |
| S199 | | 8 | 1 | 0 | - | |
| N200 | | 16 | 6 | 0 | - | |
| S201 | | 8 | 0 | 0 | - | |
| V202 | | 0 | 0 | 0 | - | |
| Y203 | | 8 | 8 | 7 | No | |
| I204 | | 8 | 0 | 0 | - | |
| T205 | | 8 | 6 | 5 | No | |
| L206 | | 12 | 4 | 3 | Yes | L206G |
| F207 | | 8 | 5 | 0 | - | |
| K208 | | 8 | 1 | 0 | - | |
| T209 | | 16 | 5 | 1 | No | |
| M210 | | 16 | 8 | 1 | No | |

| Amino acid | TM prediction (TMHMM) | Number of colonies assayed for growth and whole-cell fluorescence | Initial hits ($OD_{600}$ 16 µg/ml/0 µg.ml tetracycline <0.14) | Reproducibly not tetracycline resistant | In-gel fluorescence at TetA(B)-TEV-His10-eGFP size | Sequence of transport-compromised mutants |
|---|---|---|---|---|---|---|
| P211 | | 8 | 1 | 0 | - | |
| I212 | 7 | 8 | 0 | 0 | - | |
| L213 | 7 | 8 | 1 | 0 | - | |
| L214 | 7 | 8 | 8 | 1 | Yes | L214G |
| I215 | 7 | 8 | 7 | 0 | - | |
| I216 | 7 | 8 | 0 | 0 | - | |
| Y217 | 7 | 8 | 8 | 0 | - | |
| F218 | 7 | 8 | 0 | 0 | - | |
| S219 | 7 | 8 | 1 | 0 | - | |
| A220 | 7 | 8 | 0 | 0 | - | |
| Q221 | 7 | 4 | 4 | 3 | Yes x2 | Not correct vector |
| L222 | 7 | 8 | 3 | 1 | No | |
| I223 | 7 | 8 | 0 | 0 | - | |
| G224 | 7 | 8 | 1 | 0 | - | |
| Q225 | 7 | 16 | 3 | 0 | - | |
| I226 | 7 | 8 | 0 | 0 | - | |
| P227 | 7 | 8 | 1 | 1 | 1 | P227V |
| A228 | 7 | 0 | 0 | 0 | - | |
| T229 | 7 | 8 | 1 | 0 | - | |
| V230 | 7 | 8 | 0 | 0 | - | |
| W231 | 7 | 8 | 0 | 0 | - | |
| V232 | 7 | 8 | 2 | 2 | No | |
| L233 | 7 | 4 | 3 | 2 | No | |
| F234 | 7 | 9 | 5 | 4 | Yes x2 | F234A |
| T235 | | 8 | 0 | 0 | - | |
| E236 | | 12 | 9 | 4 | No | |
| N237 | | 8 | 2 | 0 | - | |
| R238 | | 8 | 1 | 0 | - | |
| F239 | | 8 | 1 | 1 | Yes | R127G + I143V |
| G240 | | 8 | 4 | 4 | No | |
| W241 | | 8 | 2 | 2 | No | |
| N242 | | 8 | 8 | 2 | Yes | N242A + Q319H |
| S243 | | 8 | 3 | 2 | No | |
| M244 | 8 | 8 | 0 | 0 | - | |
| M245 | 8 | 8 | 4 | 3 | No | |
| V246 | 8 | 8 | 6 | 4 | No | |
| G247 | 8 | 8 | 3 | 0 | No | |
| F248 | 8 | 8 | 3 | 1 | No | |
| S249 | 8 | 8 | 1 | 0 | - | |
| L250 | 8 | 8 | 0 | 0 | - | |
| A251 | 8 | 8 | 1 | 0 | - | |
| G252 | 8 | 1 | 1 | 0 | - | |
| L253 | 8 | 8 | 3 | 0 | - | |

| Amino acid | TM prediction (TMHMM) | Number of colonies assayed for growth and whole-cell fluorescence | Initial hits ($OD_{600}$ 16 µg/ml/0 µg.ml tetracycline <0.14) | Reproducibly not tetracycline resistant | In-gel fluorescence at TetA(B)-TEV-His10-eGFP size | Sequence of transport-compromised mutants |
|---|---|---|---|---|---|---|
| G254 | 8 | 8 | 1 | 0 | - | |
| L255 | 8 | 8 | 2 | 1 | No | |
| L256 | 8 | 8 | 2 | 1 | No | |
| H257 | 8 | 8 | 4 | 0 | - | |
| S258 | 8 | 8 | 1 | 0 | - | |
| V259 | 8 | 8 | 1 | 0 | - | |
| F260 | 8 | 8 | 0 | 0 | - | |
| Q261 | 8 | 13 | 8 | 5 | Yes | Q261V |
| A262 | 8 | 2 | 2 | 0 | - | |
| F263 | 8 | 8 | 1 | 0 | - | |
| V264 | 8 | 8 | 1 | 0 | - | |
| A265 | 8 | 11 | 2 | 0 | - | |
| G266 | 8 | 8 | 6 | 1 | Yes | Not correct vector |
| R267 | | 8 | 3 | 1 | No | |
| I268 | | 0 | 0 | 0 | No | |
| A269 | | 8 | 3 | 0 | - | |
| T270 | | 8 | 0 | 0 | - | |
| K271 | | 8 | 1 | 0 | - | |
| W272 | | 4 | 2 | 0 | - | |
| G273 | | 8 | 0 | 0 | - | |
| E274 | | 8 | 0 | 0 | - | |
| K275 | | 8 | 0 | 0 | - | |
| T276 | | 8 | 6 | 6 | No | |
| A277 | | 8 | 0 | 0 | - | |
| V278 | 9 | 8 | 0 | 0 | - | |
| L279 | 9 | 8 | 0 | 0 | - | |
| L280 | 9 | 8 | 1 | 0 | - | |
| G281 | 9 | 8 | 4 | 2 | No | |
| F282 | 9 | 8 | 0 | 0 | - | |
| I283 | 9 | 8 | 0 | 0 | - | |
| A284 | 9 | 8 | 0 | 0 | - | |
| D285 | 9 | 8 | 6 | 4 | Yes x4 | D285A, D285G and D285V |
| S286 | 9 | 8 | 0 | 0 | - | |
| S287 | 9 | 8 | 0 | 0 | - | |
| A288 | 9 | 8 | 0 | 0 | - | |
| F289 | 9 | 8 | 0 | 0 | - | |
| A290 | 9 | 8 | 0 | 0 | - | |
| F291 | 9 | 8 | 0 | 0 | - | |
| L292 | 9 | 8 | 6 | 0 | - | |
| A293 | 9 | 8 | 4 | 2 | No | |
| F294 | 9 | 8 | 1 | 0 | - | |
| I295 | 9 | 8 | 1 | 0 | - | |
| S296 | | 8 | 0 | 0 | - | |
| E297 | | 8 | 0 | 0 | - | |

| Amino acid | TM prediction (TMHMM) | Number of colonies assayed for growth and whole-cell fluorescence | Initial hits ($OD_{600}$ 16 µg/ml/0 µg.ml tetracycline <0.14) | Reproducibly not tetracycline resistant | In-gel fluorescence at TetA(B)-TEV-His10-eGFP size | Sequence of transport-compromised mutants |
|---|---|---|---|---|---|---|
| G298 | | 8 | 0 | 0 | - | |
| W299 | 10 | 16 | 0 | 0 | - | |
| L300 | 10 | 8 | 0 | 0 | - | |
| V301 | 10 | 8 | 0 | 0 | - | |
| F302 | 10 | 8 | 2 | 1 | No | |
| P303 | 10 | 8 | 1 | 0 | - | |
| V304 | 10 | 8 | 3 | 3 | No | |
| L305 | 10 | 8 | 1 | 0 | - | |
| I306 | 10 | 8 | 6 | 4 | No | |
| L307 | 10 | 8 | 0 | 0 | - | |
| L308 | 10 | 8 | 5 | 2 | Yes x2 | L308G |
| A309 | 10 | 16 | 3 | 1 | Yes | A309V |
| G310 | 10 | 8 | 1 | 0 | - | |
| G311 | 10 | 8 | 4 | 1 | No | |
| G312 | 10 | 8 | 1 | 0 | - | |
| I313 | 10 | 8 | 3 | 1 | No | |
| A314 | 10 | 8 | 1 | 0 | - | |
| L315 | 10 | 8 | 0 | 0 | - | |
| P316 | 10 | 8 | 8 | 7 | No | |
| A317 | 10 | 8 | 1 | 0 | - | |
| L318 | 10 | 8 | 1 | 1 | No | |
| Q319 | 10 | 8 | 1 | 0 | - | |
| G320 | 10 | 8 | 4 | 1 | No | |
| V321 | 10 | 8 | 2 | 1 | No | |
| M322 | | 8 | 2 | 0 | - | |
| S323 | | 8 | 6 | 0 | - | |
| I324 | | 8 | 0 | 0 | - | |
| Q325 | | 16 | 1 | 0 | - | |
| T326 | | 8 | 0 | 0 | - | |
| K327 | | 8 | 0 | 0 | - | |
| S328 | | 8 | 0 | 0 | - | |
| H329 | | 8 | 0 | 0 | - | |
| Q330 | | 8 | 0 | 0 | - | |
| Q331 | | 8 | 0 | 0 | - | |
| G332 | | 8 | 0 | 0 | - | |
| A333 | | 8 | 0 | 0 | - | |
| L334 | 11 | 8 | 0 | 0 | - | |
| Q335 | 11 | 8 | 1 | 1 | Yes | Q335V |
| G336 | 11 | 8 | 3 | 2 | Yes x2 | G336V |
| L337 | 11 | 8 | 0 | 0 | - | |
| L338 | 11 | 8 | 0 | 0 | - | |
| V339 | 11 | 8 | 0 | 0 | - | |
| S340 | 11 | 8 | 0 | 0 | - | |

| Amino acid | TM prediction (TMHMM) | Number of colonies assayed for growth and whole-cell fluorescence | Initial hits ($OD_{600}$ 16 µg/ml/0 µg.ml tetracycline <0.14) | Reproducibly not tetracycline resistant | In-gel fluorescence at TetA(B)-TEV-His10-eGFP size | Sequence of transport-compromised mutants |
|---|---|---|---|---|---|---|
| L341 | 11 | 8 | 1 | 0 | - | |
| T342 | 11 | 8 | 0 | 0 | - | |
| N343 | 11 | 8 | 1 | 1 | Yes | N343V |
| A344 | 11 | 8 | 0 | 0 | - | |
| T345 | 11 | 8 | 0 | 0 | - | |
| G346 | 11 | 8 | 3 | 2 | Yes x2 | G346V |
| V347 | 11 | 8 | 1 | 1 | No | |
| I348 | 11 | 8 | 0 | 0 | - | |
| G349 | 11 | 8 | 0 | 0 | - | |
| P350 | 11 | 8 | 0 | 0 | - | |
| L351 | 11 | 8 | 0 | 0 | - | |
| L352 | 11 | 8 | 0 | 0 | - | |
| F353 | 11 | 8 | 0 | 0 | - | |
| A354 | 11 | 8 | 1 | 0 | - | |
| V355 | 11 | 8 | 0 | 0 | - | |
| I356 | 11 | 8 | 0 | 0 | - | |
| Y357 | | 8 | 1 | 0 | - | |
| N358 | | 8 | 1 | 0 | - | |
| H359 | | 8 | 1 | 0 | - | |
| S360 | | 8 | 0 | 0 | - | |
| L361 | | 8 | 2 | 0 | - | |
| P362 | | 8 | 4 | 0 | - | |
| I363 | | 8 | 3 | 0 | - | |
| W364 | | 8 | 5 | 0 | - | |
| D365 | | 8 | 0 | 0 | - | |
| G366 | 12 | 8 | 5 | 0 | - | |
| W367 | 12 | 8 | 0 | 0 | - | |
| I368 | 12 | 8 | 0 | 0 | - | |
| W369 | 12 | 8 | 3 | 1 | Yes | W369G |
| I370 | 12 | 8 | 1 | 0 | - | |
| I371 | 12 | 8 | 0 | 0 | - | |
| G372 | 12 | 8 | 0 | 0 | - | |
| L373 | 12 | 8 | 0 | 0 | - | |
| A374 | 12 | 8 | 0 | 0 | - | |
| F375 | 12 | 8 | 0 | 0 | - | |
| Y376 | 12 | 8 | 0 | 0 | - | |
| C377 | 12 | 8 | 1 | 1 | No | |
| I378 | 12 | 8 | 0 | 0 | - | |
| I379 | 12 | 8 | 0 | 0 | - | |

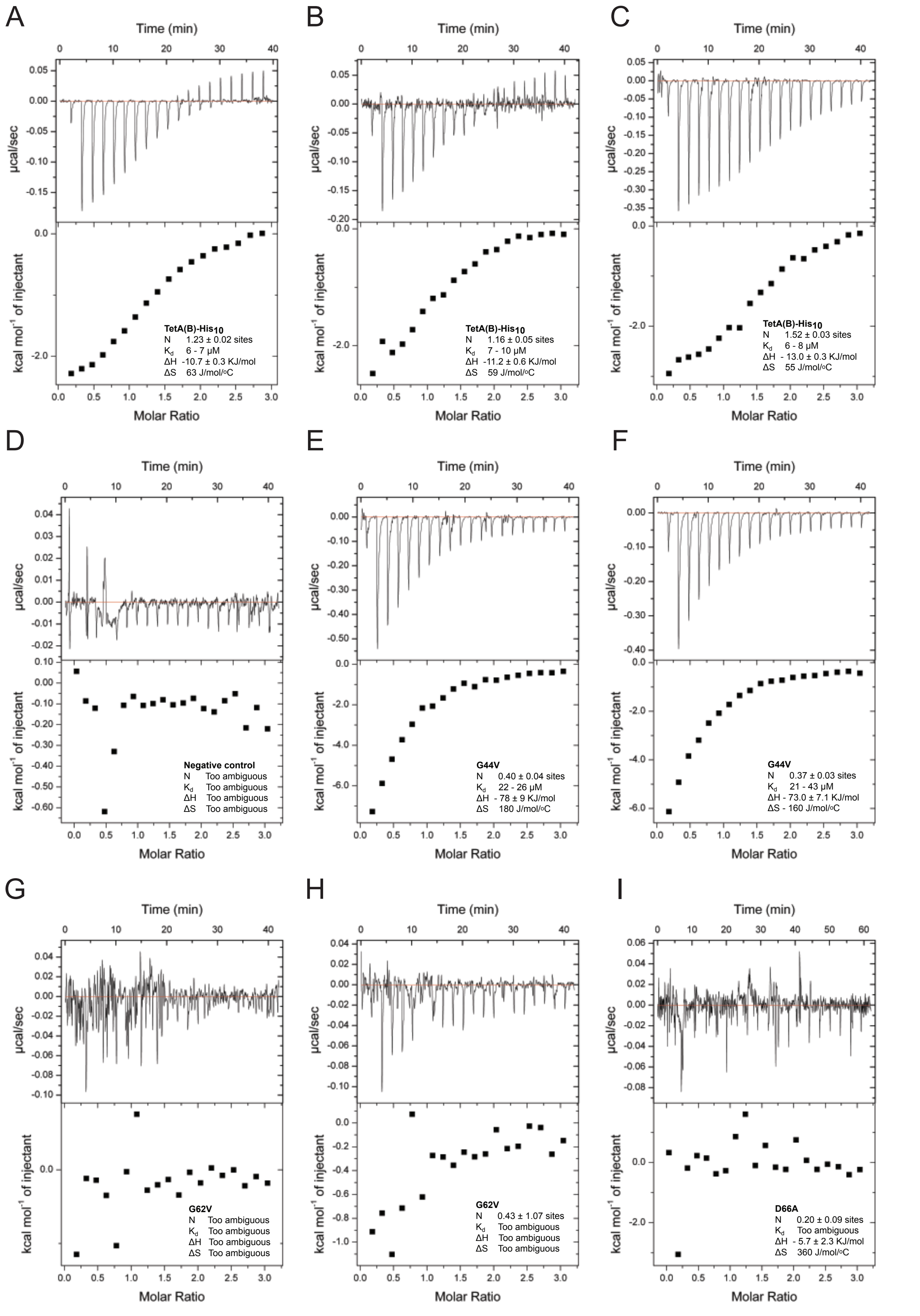


**Supplementary Figure 1** ITC data for TetA(B) mutants. All mutants were constructed in TetA(B)-His10 and experiments were performed in either duplicate or triplicate. A-C, TetA(B)-His10; D, negative control; E, F, G44V; G, H, G62V; I, D66A.

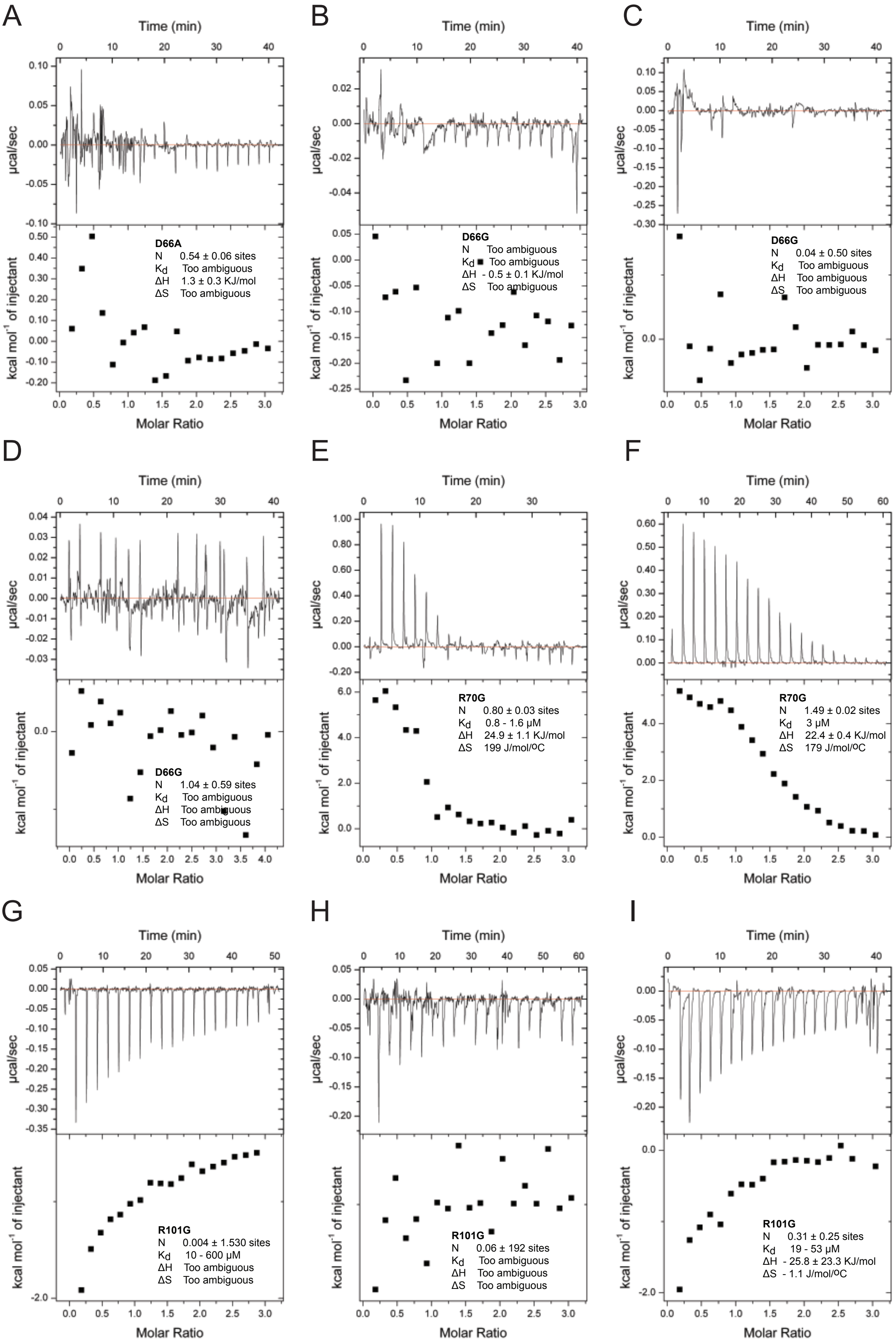


**Supplementary Figure 2** ITC data for TetA(B) mutants. All mutants were constructed in TetA(B)-His10 and experiments were performed in either duplicate or triplicate. A, D66A; B-D, D66G; E, F, R70G; G-I, R101G.

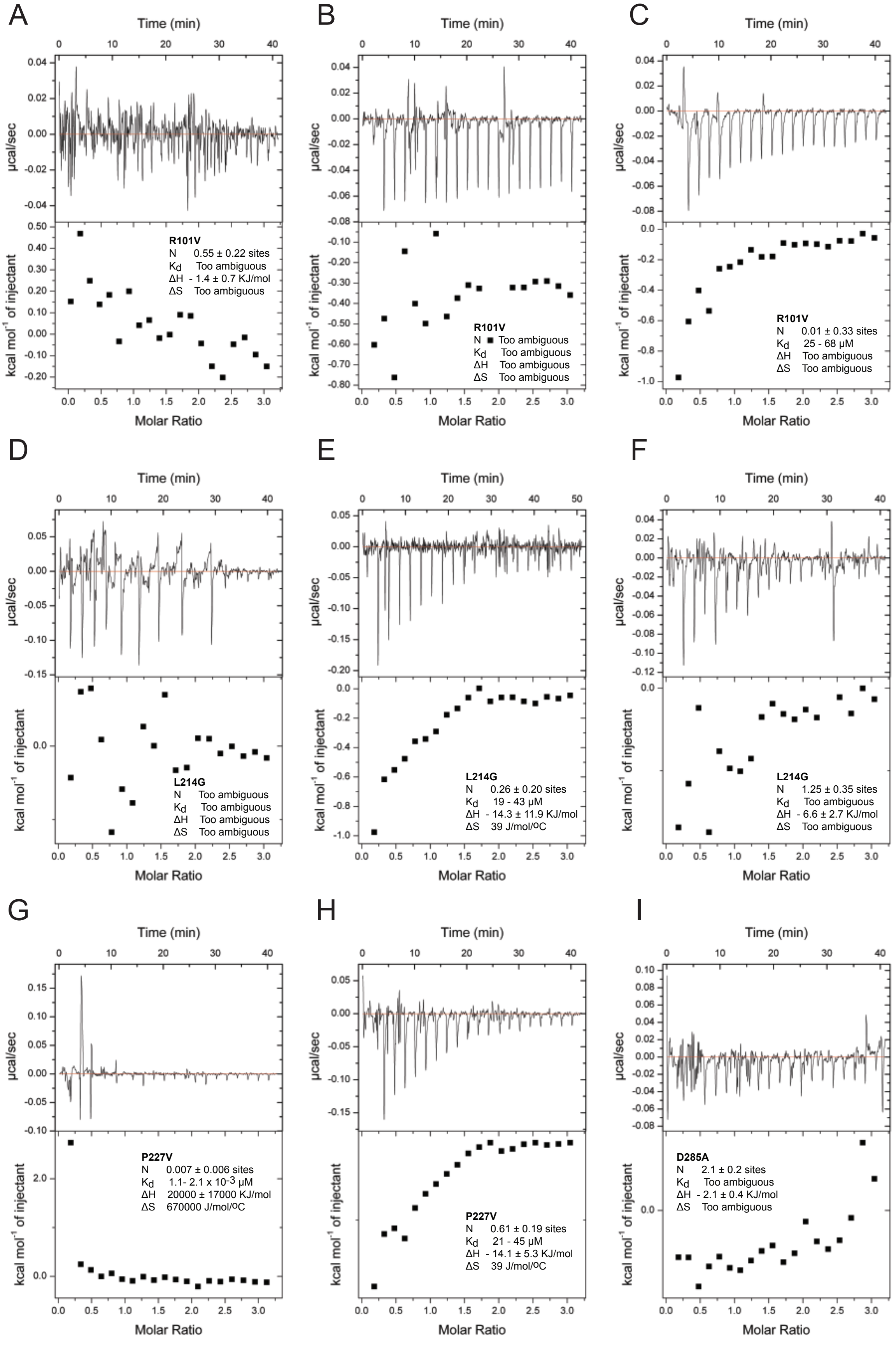


**Supplementary Figure 3** ITC data for TetA(B) mutants. All mutants were constructed in TetA(B)-His10 and experiments were performed in either duplicate or triplicate. A-C, R101V; D-F, L214G; G, H, P227V; I, D285A.

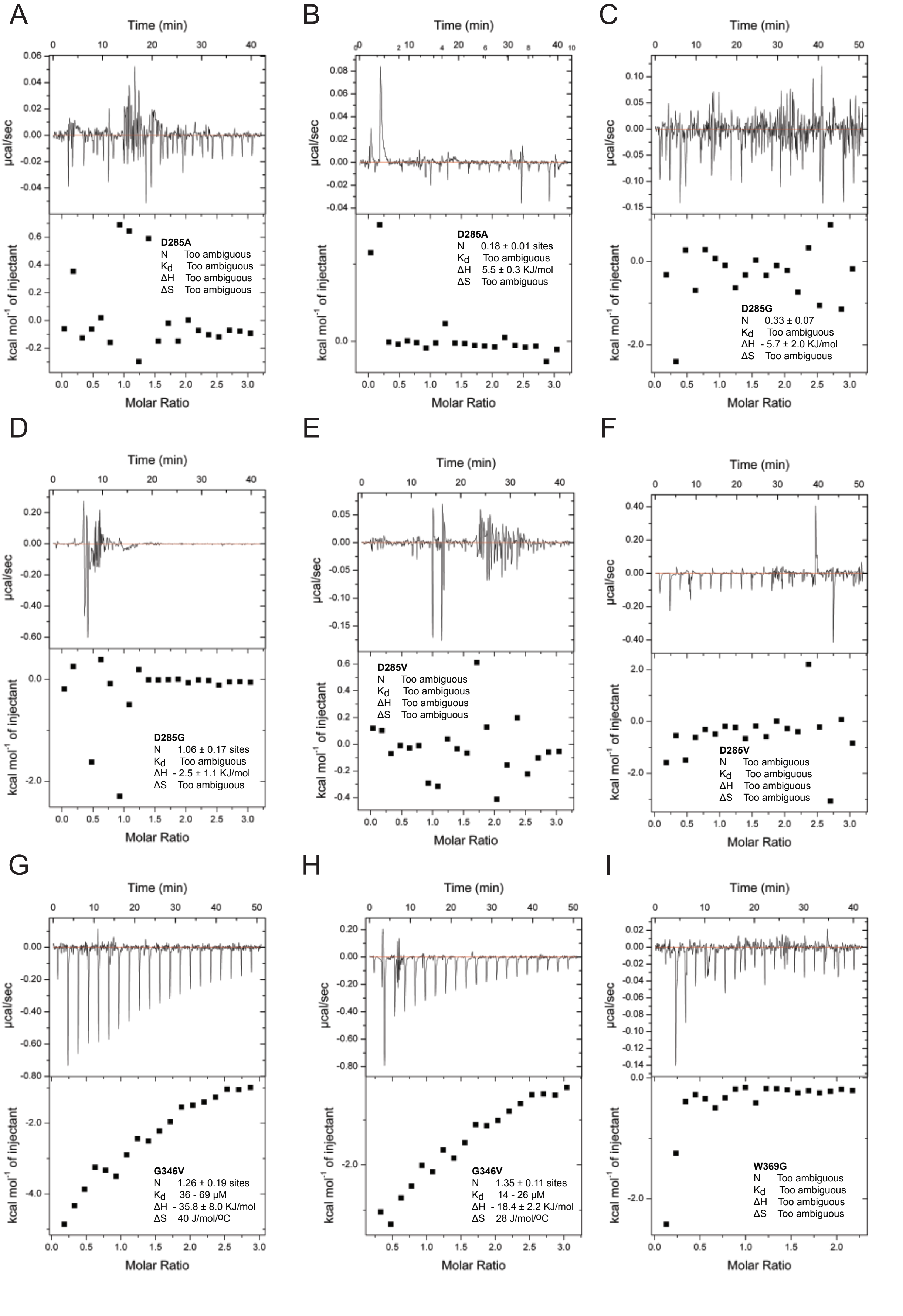


**Supplementary Figure 4** ITC data for TetA(B) mutants. All mutants were constructed in TetA(B)-His10 and experiments were performed in either duplicate or triplicate. A,B, D285A; C,D, D285G; E, F, D285V; G, H, G346V; I, W369G.

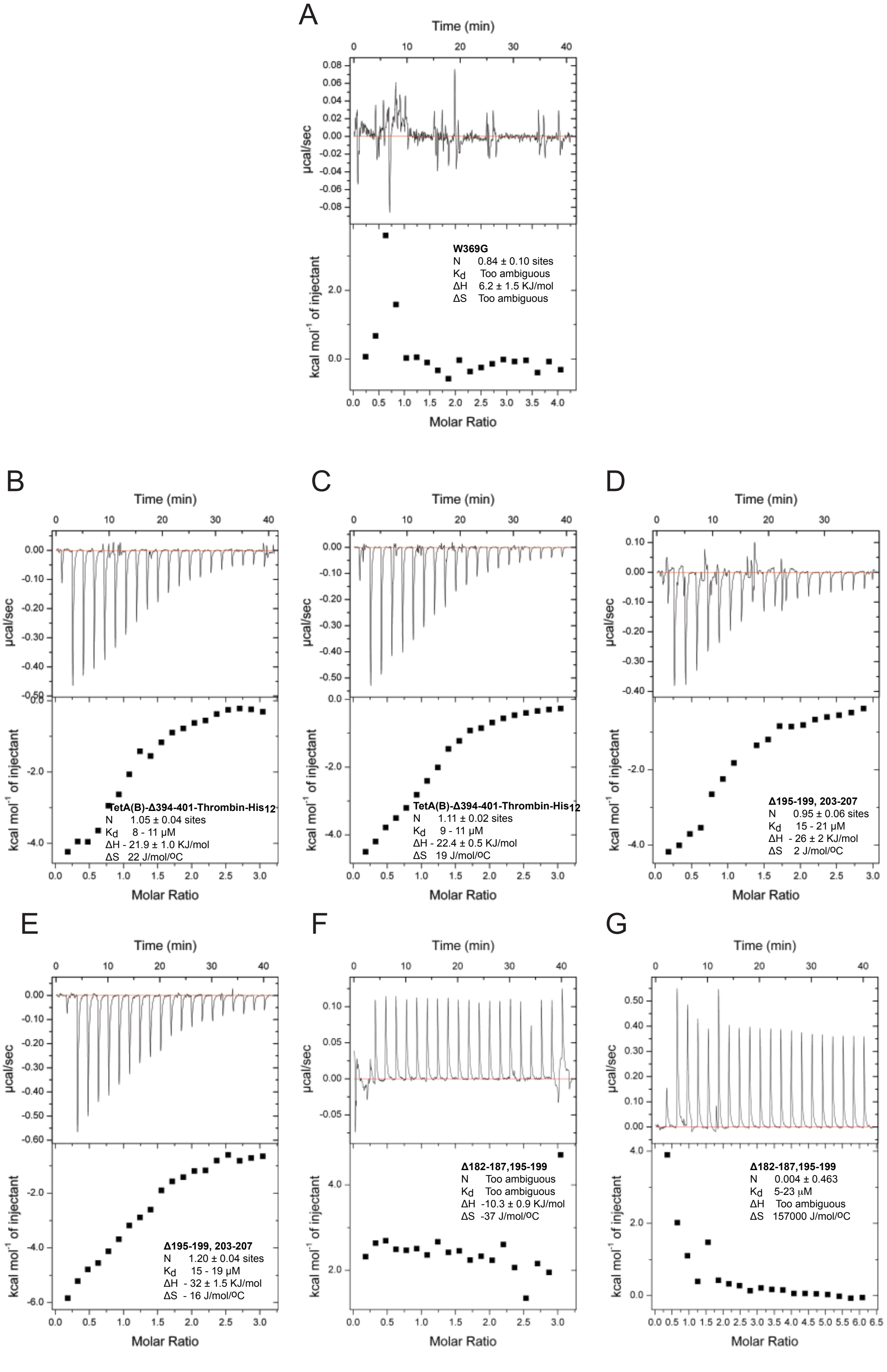


**Supplementary Figure 5** ITC data for TetA(B) mutants. Point mutants were constructed in TetA(B)-His10 whereas the loop deletion mutants were constructed in TetA(B)-Δ394-401-Th-His12. Experiments were performed in either duplicate or triplicate.
A, W369G; B,C, positive control, TetA(B)-D394-401-Th-His12; D,E, Δ195-199,203-207; F,G, Δ182-187,195-199.

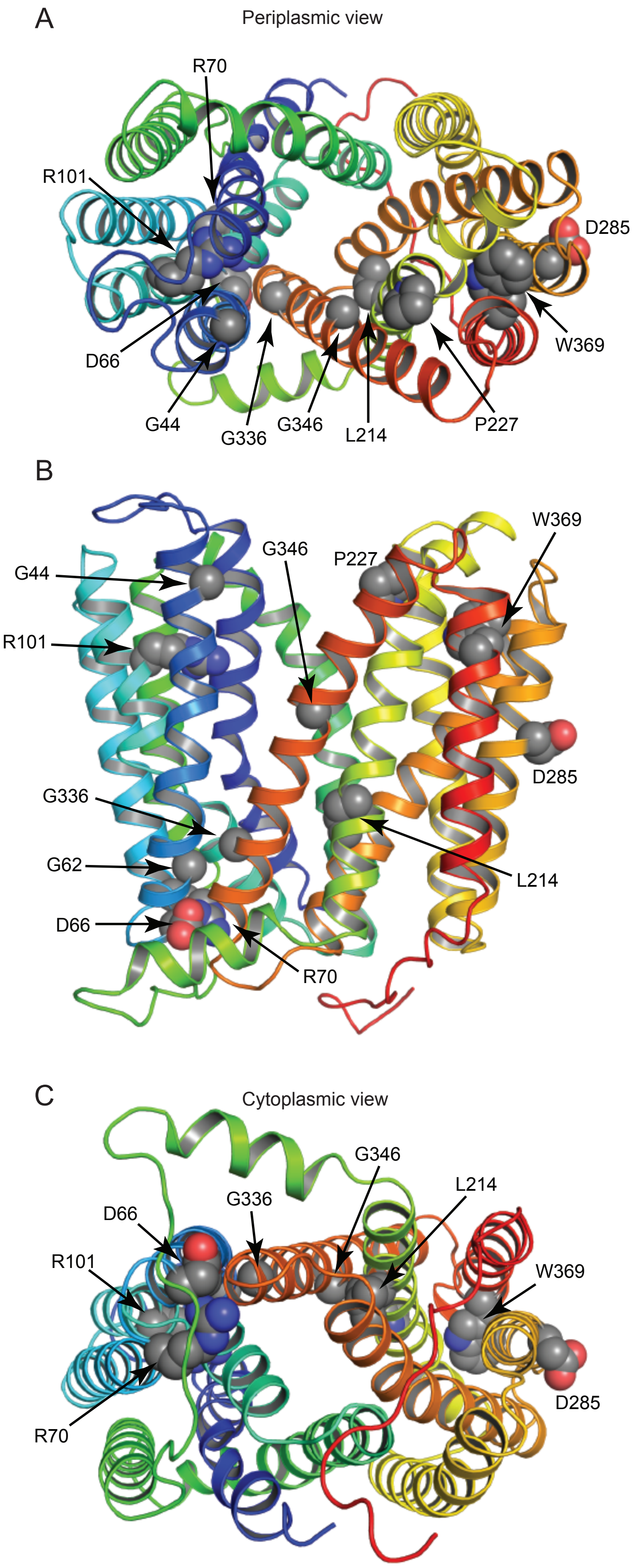


**Supplementary Figure 6.** Model of TetA(B) highlighting the putative positions of residues that, when mutated, affect the ability of the mutant to transport tetracycline. The model is depicted as a cartoon in rainbow colouration (N terminus blue, C terminus red) with side chains depicted as space-filling models (carbon, grey; nitrogen, blu; oxygen, red). A, view from the periplasm; B, view from in the plane of the lipid bilayer; C, view from the cytoplasm.
